# Identification of novel reassortant mammalian orthoreoviruses from bats in Slovenia

**DOI:** 10.1186/s12917-018-1585-y

**Published:** 2018-09-03

**Authors:** Tina Naglič, Danijela Rihtarič, Peter Hostnik, Nataša Toplak, Simon Koren, Urška Kuhar, Urška Jamnikar-Ciglenečki, Denis Kutnjak, Andrej Steyer

**Affiliations:** 1 Institute of Microbiology and Immunology, Faculty of Medicine, University of Ljubljana, Zaloška cesta 4, SI-1000 Ljubljana, Slovenia; 2 Institute of Microbiology and Parasitology, Veterinary Faculty, University of Ljubljana, Gerbičeva ulica 60, Ljubljana, Slovenia; 3 Omega d.o.o, Dolinškova ulica 8, Ljubljana, Slovenia; 4 Institute of Food Safety, Feed and Environment, Veterinary Faculty, University of Ljubljana, Gerbičeva ulica 60, Ljubljana, Slovenia; 5 National Institute of Biology, Večna pot, 111 Ljubljana, Slovenia

**Keywords:** Bats, Genome reassortment, Mammalian orthoreovirus, Whole genome sequencing

## Abstract

**Background:**

Recently, mammalian orthoreoviruses (MRVs) were detected for the first time in European bats, and the closely related strain SI-MRV01 was isolated from a child with severe diarrhoea in Slovenia. Genetically similar strains have also been reported from other mammals, which reveals their wide host distribution. The aim of this study was to retrospectively investigate the occurrence and genetic diversity of MRVs in bats in Slovenia, from samples obtained throughout the country in 2008 to 2010, and in 2012 and to investigate the occurrence of the novel SI-MRV01 MRV variant in Slovenian bats.

**Results:**

The detection of MRVs in bat guano was based on broad-range RT-PCR and specific bat MRV real-time RT-PCR. Subsequently, MRV isolates were obtained from cell culture propagation, with detailed molecular characterisation through whole-genome sequencing.

Overall, bat MRVs were detected in 1.9% to 3.8% of bats in 2008, 2009 and 2012. However, in 2010 the prevalence was 33.0%, which defined an outbreak of the single SI-MRV01 strain. Here, we report on the identification of five MRV isolates of different serotypes that are designated as SI-MRV02, SI-MRV03, SI-MRV04, SI-MRV05 and SI-MRV06. There is high genetic variability between these characterised isolates, with evident genome reassortment seen across their genome segments.

**Conclusions:**

In conclusion, we have confirmed the presence of the SI-MRV01 strain in a Slovenian bat population. Moreover, according to genetic characterisation of S1 genome segment, all three MRV serotypes were present in the bat population. In this study, five independent MRV isolates were obtained and detailed whole genome analysis revealed high diversity between them. This study generates new information about the epidemiology and molecular characteristics of emerging bat MRV variants, and provides important molecular data for further studies of their pathogenesis and evolution.

**Electronic supplementary material:**

The online version of this article (10.1186/s12917-018-1585-y) contains supplementary material, which is available to authorized users.

## Background

Mammalian orthoreoviruses (MRVs) are type species of the genus *Orthoreovirus*, subfamily *Spinareovirinae*, family *Reoviridae* and can infect nearly all mammals [1]. Since the first description of MRVs in the 1950s [2], they have been thoroughly studied not only from the genetics and structure perspectives, but also in terms of their epidemiology and pathogenicity [1]. They were initially isolated from the respiratory and gastrointestinal tracts in humans, but were rarely associated with severe medical conditions [2]. In the past few years, there have been several reports of novel MRV variants that can cause severe illness, such as haemorrhagic enteritis, upper respiratory tract infections and encephalitis, in humans and other animals [3, 4].

The MRV genome contains 10 dsRNA segments that are designated as the large (L, three segments), medium (M, three segments) and small (S, four segments) segments, based on their electrophoretic mobilities [5]. Neutralisation and haemagglutinin activities are restricted to the S1 gene segment [6], which encodes the σ1 protein that is located on the outer capsid of the virion. The σ1 protein is responsible for viral attachment to cellular receptors, and it defines the MRV serotype [7]. The other genome segments show no correlations to viral serotype, which suggests that MRVs evolved independently of their serotypes [8]. The segmented nature of the MRV genome poses risks for the formation of novel reassortant viruses with unpredictable biological properties. Indeed, isolation of reassortant MRVs has been described previously [9–13].

In our previous study, a distinct MRV strain (SI-MRV01) was detected in 2012 in a child hospitalised for severe gastroenteritis, with some further symptoms, including red and swollen gums, with oral ulcers [12]. The genome nucleotide sequence of isolate SI-MRV01 shared more than 97% identity with MRVs that were isolated at the same time from bats of the genera *Pipistrellus* spp. and *Myotis* spp. in Italy and Germany [14, 15]. These were the first reports of non-pteropine MRVs in bats. Both of these bat genera are also present in Slovenia. Isolate SI-MRV01 was also the first reported bat MRV variant found in humans. More recently, a MRV with high similarity to SI-MRV01 was detected in an immunocompromised child in Switzerland [16]. Based on the literature data, we consider that isolate SI-MRV01 and other similar isolates represent novel zoonotic MRV variants that are present in European bats. To date, the biological characteristics of this virus have not been investigated in detail.

Bats are the most abundant, assorted and geographically dispersed vertebrates, and they are increasingly known as reservoirs of viruses that can cross species barriers to infect other domestic and wild animals, and humans [17]. Human intervention in nature can lead to interaction with bats, which could be source of infection with emerging pathogens. In Slovenia, there are 30 insectivorous bat species [18]. Recently, detection of virus species in bats has increased rapidly not only due to the development of suitable molecular methods but also due to increasing sampling effort and interest in finding different viruses with zoonotic potential.

The aim of the present study was to retrospectively investigate the occurrence and genetic diversity of MRVs in bats in Slovenia, from samples obtained throughout the country in 2008 to 2010, and in 2012. Our aim was also to investigate the occurrence of the novel SI-MRV01 MRV variant in Slovenian bats, as this might represent the source of infection for humans. An important goal of the present study was also to obtain infectious virus isolates in cell culture for further studies on virus biology. In summary, this study generates new information about the epidemiology and molecular characteristics of emerging bat MRV variants, and provides important molecular data for further studies of their pathogenesis and evolution.

## Results

### MRV positives

In total, 44 out of 443 individual guano samples (9.9%) were positive for MRV RNA, regardless of the molecular screening method (Table 1). Among the positive samples, there was one pooled sample from a lactating female and a juvenile male (See Additional file 3). The prevalence of MRV RNA in sampling year 2010 stood out in particular, and this was over 10-fold higher than for the other three sampling years, at 33.0% (Table 2). Moreover, all of the 15 samples in 2010 in which the partial MRV L3 genome segment was sequenced shared 99% nucleotide sequence identity, and highest similarity with strain, SI-MRV01, a bat MRV isolated from a child with severe gastroenteritis in Slovenia in 2012 [12].

**Table 1:** Bat characteristics of the individual guano samples

Gender	MRV RNA positive samples/Bats tested [n/n(%)]				
	Total	Juvenile	Adult	Lactating female	Gravid female
Female	29/268 (10.8)	9/72 (12.5)	1/36 (2.8)	19/141 (13.5)	0/19 (0)
Male	13/129 (10.1)	3/30 (10.0)	10/99 (10.1)	na	na
Unknown	2/46 (4.3)	na	na	na	na
Total	44/443 (9.9)	12/102 (11.8)	11/135 (8.1)	19/141 (13.5)	0/19

*na* not applicable

**Table 2:** Characteristics of MRV RNA positive guano samples

Year	Samples	MRV RNA		Bat species positive for MRV RNA (*n*)
	tested (*n*)	Positives (real-time RT-PCR/RT-PCR)	Prevalence (%)	
2008	108	2 (0/2)	1.9	*Rhinolophus hipposideros* (1), *Myotis myotis* (1)
2009	186	7 (7/3)	3.8	*Myotis emarginatus* (3), *Eptesicus serotinus* (2), *Myotis daubentonii* (1), unknown (1)
2010	103	34 (34/15)	33.0	*Eptesicus serotinus* (18), *Miniopterus schreibersii* (8), *Myotis myotis* (4), *Myotis daubentonii* (2), *Rhinolophus hipposideros* (1), unknown (1)
2012	54	2 (0/2)	3.7	*Myotis daubentonii* (2)

The most numerous bat species that was positive for MRV RNA in 2010 was the serotine bat, *Eptesicus serotinus*, as 18 positive out of the 94 tested. Overall, six bat species were positive for MRV RNA across all of the sampled years (Table 1). Based on gender, age and lactation status, the more highly positive samples were from lactating females (19 positive of 141 individual samples tested), followed by adult males (10 positive out of 99 individual samples tested). Nine samples were from juvenile females, three from juvenile males, and one from an adult female. Two MRV RNA positive samples were from samples of unknown origin (Table 1).

Statistical analysis showed association between MRV RNA presence in guano samples and sampling years (*p* = 0.0023; OR = 1.44; 95% CI 1.14, 1.82). The association between MRV RNA presence in guano samples and bat gender, age, species and sampling region was not significant.

### MRV isolates

From 45 MRV RNA positive guano samples, nine were selected for virus isolation by cell culture. This virus isolation was successful from five guano samples, where the cytopathic effects developed within 3 to 8 days post inoculation. The reoviral morphology from the cell culture supernatants was confirmed under electron microscopy. The MRV isolates were denoted as SI-MRV02, SI-MRV03, SI-MRV04, SI-MRV05 and SI-MRV06 (Table 3), and were then molecularly characterised in depth, to preliminary classify the virus into serotype, based on genetic characterisation of S1 genome segment. All of the isolates characterised in this study were deposited with the European Virus Archive (EVAg; https://www.european-virus-archive.com/), and are available upon request.

**Table 3:** Characteristics of Slovenian MRV strains successfully isolated from the bats using cell culture

MRV isolate	Year	Guano sample code	Appearance of cytopathic effect		Bat host species	MRV serotype^b^	EVAg Ref. No.^a^
			Days post inoculation	Passage number			
SI-MRV02	2010	SLO1A 4566/10	3	First	*Eptesicus serotinus*	3	007 V-02717
SI-MRV03	2012	SLO1A 2361/12	5	First	*Myotis daubentonii*	1	007 V-02718
SI-MRV04	2009	SLO1A 4449/09	4	First	*Eptesicus serotinus*	1	007 V-02719
SI-MRV05	2008	SLO1A 4273/08	8	First	*Myotis myotis*	2	007 V-02720
SI-MRV06	2009	SLO1A 2200/09	3	Second	*Myotis emarginatus*	1	007 V-02721

^a^, All isolates are deposited with the European Virus Archive (EVAg; https://www.european-virus-archive.com/)

^b^, The serotype is determined by genetic characterisation of S1 genome segment

### Molecular analysis

The whole genomes of all of these five MRV isolates from bats were analysed in detail. For the whole sequences for all 10 of the genome segments, mapping to reference mammalian orthoreovirus was successful for only three of the MRV isolates: SI-MRV02, SI-MRV05 and SI-MRV06. For the other two MRV isolates, SI-MRV03 and SI-MRV04, mapping to several reference MRV isolates of different serotypes and origins did not result in whole-genome assembly. The problematic genome segment was always S1, which was subsequently assembled using the *de-novo* assembly approach, and the generated contigs were compared for similarity against all of the virus sequences deposited in the NCBI GenBank *nt* database, using BLASTn.

**Fig. 1:**
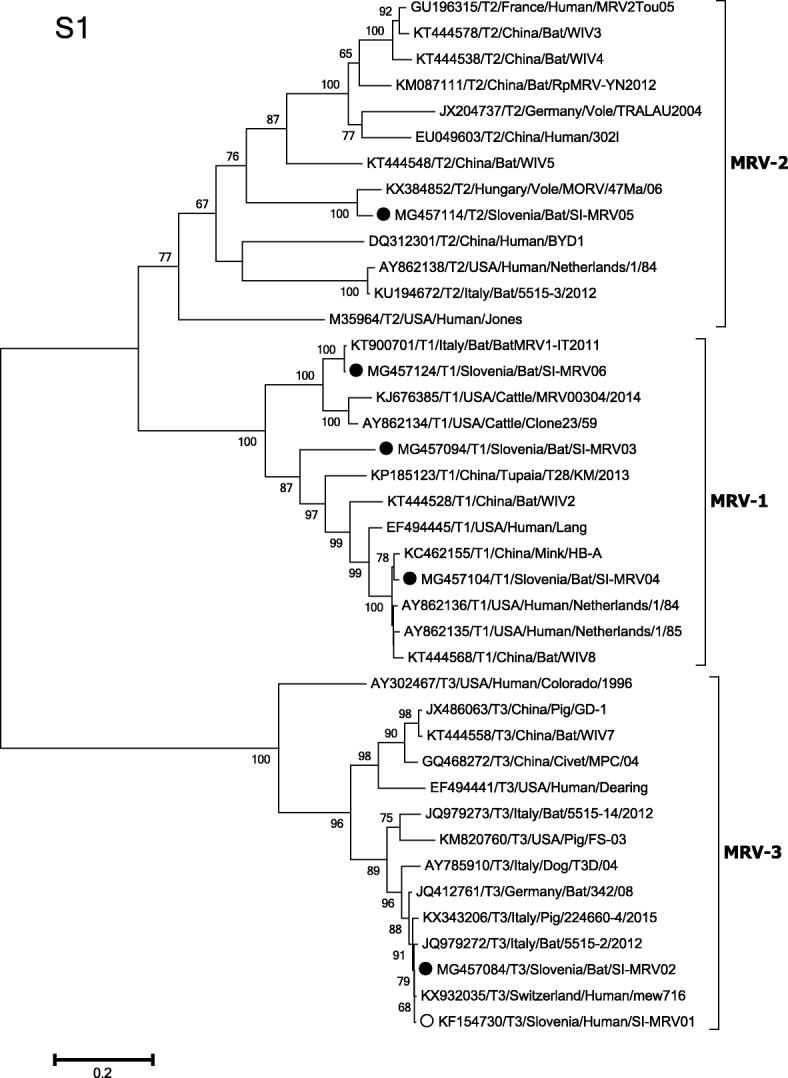
Phylogenetic tree of the 40 mammalian orthoreovirus 1389 nt ORFs of S1 genome segments. Black dots (●), label sequences of five Slovenian MRV isolates from bats from this study. White dot (○), label sequence of the Slovenian MRV isolate from a child with severe gastroenteritis [12]. The phylogenetic calculations were carried out using maximum likelihood, based on the Tamura-Nei model [24] and applying the best-fit models with 1000 bootstrap replicates. Bootstraps values < 50 are not shown. The scale bar represents the substitutions per site and is proportional to the genetic distance. Evolutionary analyses were conducted in MEGA 6.0 [23]

The most problematic isolate in terms of whole-genome assembly was isolate SI-MRV03, with only 78% nucleotide and 83% amino-acid identities to the most similar MRV isolate from NCBI GenBank, T1/T28/KM/2013 from the tupaia tree shrew in China, based on an S1 genome segment. The nucleotide and deduced amino-acid identities of the other genome segments varied from 79 to 84% and 91% to 98%, respectively, compared to the isolate with the highest similarity in GenBank (Table 4). Phylogenetic analysis for the S1 genome segment revealed that isolate SI-MRV03 clustered within MRV serotype 1 (Fig. 1). The strain SI-MRV03 was isolated from Daubentoni’s bat, *Myotis daubentonii*, in 2012.

**Table 4:** The highest nucleotide and amino-acid identity of genome ORFs of five Slovenian MRV isolates from bats compared to MRV isolates from GenBank

	SI-MRV02	Identitiy (%)		SI-MRV03	Identitiy (%)		SI-MRV04	Identitiy (%)		SI-MRV05	Identitiy (%)		SI-MRV06	Identitiy (%)	
		Nucl.	Aa		Nucl.	Aa		Nucl.	Aa		Nucl.	Aa		Nucl.	Aa
L1	SI-MRV01	99	99	5515–2/2012	82	98	5515–3/2012	99	99	BatMRV1-IT2011	99	99	BatMRV1-IT2011	99	99
L2	SI-MRV01	99	100	Neth/85	79	94	5515–3/2012	99	99	MORV/47 Ma/06	95	99	BatMRV1-IT2011	99	99
L3	SI-MRV01	99	99	224,660–4/2015	79	95	MRV2Tou05	97	99	MORV/47 Ma/06	98	99	BatMRV1-IT2011	99	99
M1	mew716_MRV-3	99	99	Netherlands 84	81	93	5515–3/2012	99	99	MORV/47 Ma/06	98	98	BatMRV1-IT2011	99	99
M2	SI-MRV01	99	99	WIV7	83	97	5515–3/2012	99	99	MORV/47 Ma/06	95	99	BatMRV1-IT2011	99	99
M3	SI-MRV01	99	100	BatMRV1-IT2011	79	91	5515–3/2012	99	99	MORV/47 Ma/06	99	99	BatMRV1-IT2011	99	99
S1	SI-MRV01	99	99	T1/T28/KM/2013	78	83	HB-A	98	99	MORV/47 Ma/06	93	95	BatMRV1-IT2011	99	99
S2	mew716_MRV-3	99	100	WIV2	81	97	5515–3/2012	99	99	MORV/47 Ma/06	97	99	BatMRV1-IT2011	99	99
S3	SI-MRV01	99	100	342/08	83	97	5515–3/2012	99	99	MORV/47 Ma/06	93	98	BatMRV1-IT2011	99	99
S4	SI-MRV01	99	100	WIV2	84	94	5515–3/2012	99	99	MORV/47 Ma/06	97	98	BatMRV1-IT2011	99	99

GenBank designations of MRV isolates are indicated

*Nucl.* nucleotide, *Aa* amino-acid

SI-MRV02 was isolated from *E. serotinus*, in 2010. The whole-genome analysis showed in 8 MRV genome segments 99% nucleotide and 99% to 100% amino-acid identities to the first of the MRV isolates here, SI-MRV01, which was detected in 2012 in the same region (Osrednjaslovenska) [12]. Other two segments, M1 and S2, showed the highest similarity to the isolate mew716_MRV-3, a MRV with high similarity to SI-MRV01, from a child with a primary immunodeficiency in Switzerland. Based on phylogenetic analysis of all MRV genome segments (see Additional file 1), the three mentioned isolates (SI-MRV01, SI-MRV02 and mew716_MRV-3) cluster in the same group in all 10 genome segments. Moreover, the nucleotide and amino-acid identities are 99–100% (Table 4). According to the sequence analysis of the short L3 region of MRV RNA positives, no other MRV isolates rather than these similar to SI-MRV01 were detected in 2010.

SI-MRV04 was also isolated from *E. serotinus*, here in 2009, and this shared 98% nucleotide and 99% amino-acid identities to the MRV isolate HB-A from mink in China, based on the S1 genome segment. According to the phylogenetic analysis of the S1 genome segment, SI-MRV04 clustered within the MRV serotype 1 group (Fig. 1). The other segments, with the exception of the L3 genome segment, shared 99% nucleotide and amino-acid identities to an Italian isolate, 5515–3/2012, from Kuhl’s pipistrelle, *Pipistrellus kuhlii*, which was identified as MRV serotype 2. Analysis of the L3 genome segment revealed 97% nucleotide and 99% amino-acid identities to a French isolate from a child with acute necrotising encephalopathy, MRV2Tou05, which clustered within the MRV serotype 2.

SI-MRV05 was isolated from a mouse-eared bat, *M. myotis*, in 2008, and it shared in all of the genome segments, except for L1, with 95% to 99% nucleotide and 98% to 99% amino-acid identities to a Hungarian isolate, MORV/47/Ma/06. This strain was isolated from the common vole, *Microtus arvalis*, and was described as a reassortant virus [13]. The BLASTn analysis of genome segment S1 revealed that the Hungarian isolate was the only score in GenBank and shared 93% nucleotide identity. According to phylogenetic analysis of genome segment S1, the isolate SI-MRV05 clustered together with the Hungarian isolate within the MRV serotype 2 group (Fig. 1). The L1 genome segment shared the highest similarity to the Italian isolate BatMRV1-IT2011 from the lesser horseshoe bat, *Rhinolophus hipposideros*, which was also the highest score in all of the genome segments for the strain SI-MRV06, which was isolated from Geoffroy’s bat, *M. emarginatus*, in 2009. Based on the whole genome analysis, isolate BatMRV1-IT2011 was recognized as a reassortant virus [11].

The nucleotide sequences obtained in this study have been deposited in GenBank under the following accession numbers: SI-MRV02, MG457078-MG457087; SI-MRV03, MG457088-MG457097; SI-MRV04, MG457098-MG457107; SI-MRV05, MG457108-MG457117; SI-MRV06, MG457118-MG457126.

## Discussion

In this study the prevalence of MRVs in Slovenian bats was investigated with particular focus on the occurrence of the novel MRV bat variant SI-MRV01, which was initially described in a child with diarrhoea [12] and represents a novel bat MRV variant. This might at least partially explain the enzootic properties of the novel MRV, as well as its zoonotic potential. Most of the MRV-positive guano samples were recorded in 2010. Moreover, all of the positives from 2010 shared 99% nucleotide identity for the partial L3 genome segment with the isolate SI-MRV01, and no scores other than isolate SI-MRV01 were obtained. This was relatively surprising for us, in terms of one year that stands out so evidently for MRV positives. This one year of high prevalence can be explained as a probable outbreak of the SI-MRV01-like isolate among the bat populations. According to our data, the outbreak was not limited to one sampling location nor to one bat species, as in 2010, positive samples were detected for eight out of 17 sampling sites across Slovenia. However, this prediction of a single-strain outbreak was based on the partial L3 genome segment analysis, which does not guarantee identical strains in all of these positive bat species. As shown in previous studies [9–13], and as also confirmed in the present study, MRVs are genetically diverse, with frequent reassortments that can result in different strain variants. Thus, this one-segment comparison between these strains might not reflect the same situation for the genome similarity in other genome segments.

The *E. serotinus*, was evidently the species that was most frequently infected with MRVs, followed by *M. daubentonii*, and *M. myotis*. Based on age, gender and lactation status, the most MRV-prevalent bat group was lactating females, with 13.5% showing positive samples, which represented 7.1% of all of the female bats included. On the contrary, the other female groups (i.e., adults, juveniles) had lower prevalence of MRV RNA, with no positives in the gestating females. The group of lactating female bats was also the largest group, as it represented approximately 50% of all of the tested female bats, and was more than the adult and juvenile male bats combined. The relative high prevalence in these lactating female bats might be due to the grouping of the lactating females in nurseries, where contact transmission of the virus might be more frequent. In the population of male bats, the most prevalent group was adult bats (10.1% MRV RNA prevalence).

In total five independent virus isolates were obtained from different bat species. The whole genome analysis revealed the relatively interesting isolate SI-MRV03, which was very different from the MRV strains in GenBank, and which shared only 73% nucleotide identity for the S1 genome segment, compared to the most similar MRV isolate T1/T28/KM/2013 from the tupaia tree shrew in China. The SI-MRV03 isolate clustered phylogenetically as a distant branch within the MRV serotype 1 clade, and is the third described serotype 1 MRV found in bats. Lelli et al. [11] previously detected a divergent serotype 1 MRV isolate BatMRV1-IT2011 in *R. hipposideros*, in the northern part of the Italy, and Yang et al. [19] described two reassortant MRV serotype 2 isolates WIV-2 and WIV-8 in Chinese *Myotis* sp. and *Hipposideros* sp., respectively. Isolate SI-MRV03 from the present study and the isolate BatMRV1-IT2011 share only 79% whole-genome identity. However, isolate SI-MRV06 from the present study, which also clusters within the MRV serotype 1 group, shares 99% whole-genome identity to the Italian bat MRV serotype 1 isolate BatMRV1-IT2011. The Italian isolate has been identified as reassortant [11]. Based on high amino-acid and nucleotide identity between isolate SI-MRV06 and the Italian isolate, we speculate that those two isolates originated from the common ancestor of reassortant MRV.

In the present study, according to genetic characterisation of S1 genome segment another preliminary MRV serotype 1 isolate was obtained, SI-MRV04, which shared 79% whole-genome identity to isolate SI-MRV03, and 87% whole-genome identity to isolates SI-MRV06 and BatMRV1-IT2011. Based on these findings, we speculate that there is evident high diversity within the MRV serotype 1 strains that circulate in bats.

Here, we also report the identification of the isolate SI-MRV05, which is, according to genetic characterisation of S1 genome segment, the third description of serotype 2 MRV in bats. Wang et al. [10] previously identified a reassortant serotype 2 MRV in the lesser horseshoe bat, *R. pusillu*, in China. Yang et al. [19] described three bat MRV serotype 2 isolates WIV-3, WIV-4 and WIV-5 in *Hipposideros* sp. in China. The SI-MRV05 isolate in the present study was most similar to the Hungarian isolate MORV/47 Ma/06 from the common vole [13], with 94.5% whole-genome identity.

The isolate SI-MRV02 was obtained in 2010, when a probable outbreak of the SI-MRV01-like isolate among the bat populations occurred. The whole genome analysis showed 99% nucleotide and 99–100% amino-acid identities in to the first of the MRV isolates here, SI-MRV01 [12] and to the isolate mew716_MRV-3, a MRV with high similarity to SI-MRV01. Based on clustering in the same phylogenetic group in all 10 MRV genome segments (see Additional file 1) and high nucleotide and amino-acid identities between these three isolates (SI-MRV01, SI-MRV02 and mew716_MRV-3), we speculate that they are sufficiently similar to originate from a common ancestor.

Considering previous reports from European groups [14, 15] and the data from the present study, we can speculate that MRV serotype 3 is the most prevalent serotype in bat species in the European bat population. However, with the detected serotypes 2 in the present study and serotype 1 in this and the study of Lelli et al. [11], the occurrence of MRVs in bats appears not to be serotype specific. However, the serotypes of the isolates in the present study were predicted through phylogenetic clustering, and so they do not reflect antigen reactivities of serotype-specific antibodies. Moreover, to the best of our knowledge, there has not been any precise characterisation study of the antigen epitopes of the S1 protein, which might make reliable serotype characterisation based on nucleotide and/or amino-acid sequence identities possible. Thus, cross-reactivities of serotype-specific antibodies should be performed in future to provide specific serotyping.

The present study of bat guano was performed retrospectively in order to monitor the period when this bat-like SI-MRV01 strain was found in a child with diarrhoea. As shown here, this strain was indeed the most prevalent in 2010, which was 2 years before its detection in this child. We would expect that the circulation of isolates similar to SI-MRV01 would also have been very common in 2012, when this SI-MRV01 infection of the child occurred. However, no positives similar to isolate SI-MRV01 were detected in the present testing of the guano samples from 2012. This might be due to the sampling of the wrong bat target population, or alternatively, the circulation of this virus in 2012 was relatively low and the infection of the child happened by chance. Another factor that might have contributed to detection of a lack of isolates similar to SI-MRV01 in 2012 was the limited number of guano samples that year, which was almost half the number of samples in each of the other 3 years considered here (i.e., 2008, 2009, 2010). However, strain SI-MRV01 might have been circulating already in some other mammals, and it might also have been transmitted to the child through close contact with other domestic animals. Indeed, the testing of domestic animals should be performed to clarify the complete enzootic situation of this and the other bat MRV strains, which will be indicative of the possible risk factors for the introduction of this virus into the human population.

Limitation of our study was uneven distribution of sampling each year, such as variation in sampling location throughout the study years, which could influence statistical analysis. Minor influence on results could present 10% of samples with unknown sampling data (month, location and bat species), which were excluded from statistical analysis. Statistical analysis of bat MRV RNA prevalence distribution between sampling regions and bats species has poor test power due to small group size.

MRV isolates from this study and their molecular characterisation provide the basis for further research into the pathogenesis and molecular epidemiology of these novel bat MRV variants. Indeed, detailed characterisation of these novel bat MRV variants is crucial for the development of diagnostic methods and potential antiviral drugs and vaccines. However, the pathogenic potential of these bat MRV variants still needs to be evaluated, at least in a laboratory mouse model, to define their tissue tropism and dissemination to other organs after local inoculation. Furthermore, early detection of any novel disease agent is essential to the control of such emerging microorganisms.

## Conclusions

In conclusion, we have confirmed the presence of the SI-MRV01 strain in a Slovenian bat population. Moreover, according to genetic characterisation of S1 genome segment, all three MRV serotypes were present in the bat population. In this study, five independent MRV isolates were obtained and detailed whole-genome analysis revealed high diversity between them. This study generates new information about the epidemiology and molecular characteristics of emerging bat MRV variants, and provides important molecular data for further studies of their pathogenesis and evolution.

## Methods

### Bat guano samples

Bat guano was obtained from the Veterinary Faculty, University of Ljubljana. Analysis of bat guano was performed as described by Rihtaric et al. [20]. Briefly, the samples were collected individually from clinically healthy bats in bat roosting sites, from May to October. The bats were sampled mostly in the last gravidity phase of female bats and in the time, when juveniles were old enough to feed by themselves. This periods differ among bat species, for example at the end of June the juveniles of *M. myotis* were quite grown-up, but the females of *R. hipposideros* were only in a phase of delivery. The bats were classified by bat biologists from Centre for Cartography Fauna and Flora, Slovenia according to species, gender, age, gravidity and lactation status, and typed morphological criteria. The bat biologist determined bat species exclusively based on morphologic criteria (without biopsy and genetic classification) [18]. Bats age was estimated based on bats weight, ulnar length and colour of the fur [21]. Altogether, there were 68 sampling sites that were distributed throughout all 8 Slovenian regions (Osrednjeslovenska, Gorenjska, Goriška, Primorska, Dolenjska, Savinjska, Podravska, Pomurska), with 18 bat species identified. The sampling sites for individual sampling could be very close together but within the same region. There might be minor differences in the individual sampling year (for instance, region Gorenjska was included only in 2009) but throughout the study all 8 regions were represented. (see Additional file 2). For 47 samples, the sampling locations and bat characteristics were not known. Altogether, 443 individual and eight pooled guano samples were analysed (Table 1, Additional file 3). The eight pooled samples consisted of two to seven individual bat samples. Hence, guano from 466 bats was included in the analysis. To carry out this sampling and the disturbance of protected bat species, a permit was obtained from the Slovenian Environment Agency (contracts no. 35701–80/2004, 35,601–35/2010–6). The samples were analysed retrospectively from the sampling years of 2008, 2009, 2010 and 2012.

### Molecular analysis

For the molecular analysis and virus isolation, the guano samples were resuspended in Eagle’s minimum essential medium and centrifuged at 12,600 x *g* for 10 min. Two-hundred microlitres of each supernatant was used for nucleic acids extraction (iPrep Virus DNA/RNA kits; Invitrogen, Thermo Fisher Scientific). Total nucleic acids were eluted in 100 μL elution buffer and were then used for two RT-PCRs: (a) for detection of various MRV strains from different host and origin: broad-spectrum RT-PCR to target the conserved region in L3 genome segment, using the L3–1 and L3–5 primers [4]; (b) for a sensitive and specific detection of the new bat MRV variant: specific real-time RT-PCR to target the region in L1 genome segment of SI-MRV01 isolate variants [15].

For the broad-spectrum RT-PCR, 2 μL RNA was mixed with 3.5 μL nuclease-free water and 0.5 μL 20 μM L3–5 primer; denaturation was at 95 °C for 5 min. Then, 19 μL of this RT-PCR mix was added to denaturated RNA, as: 5.5 μL nuclease-free water; 12.5 μL 2× reaction mix; 0.5 μL 20 μM L3–1 primer; and 0.5 μL RT/Platinum Taq Mix (SuperScript One-Step RT-PCR with Platinum Taq; Invitrogen, Thermo Fisher Scientific). The RT-PCR was carried out with an initial reverse transcription step at 45 °C for 30 min, followed by the PCR activation step at 94 °C for 5 min, 40 cycles of amplification (94 °C for 30 s; 50 °C for 30 s; 72 °C for 1 min), and final extension step for 10 min at 72 °C in a thermal cycler (GeneAmp PCR System 9700; Applied Biosystems, Thermo Fisher Scientific). For detection and sequencing, the PCR products were run on 1% agarose gels that contained 1× SYBR Safe DNA gel stain (Invitrogen, Thermo Fisher Scientific). The products were approximately 512 bp in size, and were purified (Qiaex II Gel Extraction kits; Qiagen), and sequenced through the Sanger sequencing method using BigDye Terminator v3.1 cycle sequencing reaction kits on a genetic analyser (ABI 3500; Applied Biosystem, Thermo Fisher Scientific). The sequencing was performed with the same RT-PCR forward and reverse primers. The sequences were analysed using the CLC Main Workbench 7 (Qiagen), and the contigs were uploaded to the Basic Local Alignment Search Tool (BLASTn) to determine the highest similarities to the sequences in the NCBI GenBank *nt* database.

For the a sensitive and specific detection of the new bat MRV variant, primers and the BatReo probe were used, as described by Kohl et al. [15], with minor modifications of the probe (modified BatReoProbeM 5’-6FAM-CCCAgTCgCggTCAT**T**ACCA**C**TCCg-BBQ-3′, modified positions in bold and underlined). The following real-time RT-PCR reaction was performed: 2 μL RNA was mixed with 1.35 μL nuclease-free water and denaturated at 95 °C for 5 min. Then 6.65 μL RT-PCR mix was added, which comprised 5 μL 2× Reaction Mix, 0.5 μL 10 μM BatReoF primer, 0.5 μL 10 μM BatReoR primer, 0.25 μL 10 μM BatReoProbeM, 0.4 μL Polymerase Mix (AgPath-ID One-Step RT-PCR kits; Ambion, Thermo Fisher Scientific). The real-time RT-PCR was carried out with an initial reverse transcription step at 45 °C for 10 min, followed by the PCR activation step at 95 °C for 10 min, and 45 cycles of amplification (95 °C for 15 s, 60 °C for 45 s). Real-time amplification was run on a StepOne Real-Time PCR system (Applied Biosystems; Thermo Fisher Scientific).

### Virus isolation

The isolation of the viruses from selected positive samples was performed using the LLC-MK2 cell line (kidney cells from rhesus monkey, *Macaca mulatta*). The guano samples for virus isolation were selected based on the following criteria: (i) sequences of partial L3 genome segment in BLASTn scored as an MRV strain, or bat MRV-specific real-time RT-PCR resulted in cycle threshold (Ct) value < 34; (ii) if more samples scored as the same MRV strain (i.e., nearly 100% nucleotide sequence similarity), only a few guano samples were selected for the virus isolation. Guano suspensions were vortexed and centrifuged at 9000 x *g* for 10 min to obtain clear supernatants. From these supernatants, 150 μL was transferred to 1.5 mL Eagle’s minimum essential medium. The inoculum was passed through 0.2 μm filters and transferred to an 80% confluent cell monolayer. The cells were incubated for 1 h at 37 °C and 5% CO_2_, to allow the binding of the viruses to the cells. After this incubation, the inoculum was discarded from the cell layer, and 7 mL Eagle’s minimum essential medium with 10% foetal bovine serum was added. The cells were further incubated under conditions specified above, and observed daily for the development of the cytopathic effect, for 14 days. After the onset of the cytopathic effect, the viruses were harvested as follows: after two freeze/ thaw cycles of the infected cell cultures and centrifugation of the cell debris, the total virus was harvested and stored at − 80 °C. In the absence of the cytopathic effect, the cryolysates were sub-cultured twice onto fresh monolayers. Virus isolates from cell cultures were examined under electron microscopy after negative staining with 2% phosphotungstic acid (pH 4.5). Electron micrograph grids were screened at 120 kV in a transmission electron microscope (JEM 1400 Plus; Jeol, Tokyo, Japan). Viral particles were identified based on their morphological characteristics. For safety reasons, handling with bat guanos and propagation of viruses isolated from bat guanos was performed at Biosafety Level 3 at the Institute of Microbiology and Immunology (Faculty of Medicine, University of Ljubljana).

### Complete genome sequencing

The whole genomes of virus isolates were determined using next-generation sequencing on the Ion Torrent PGM platform, as described by Steyer et al. [12] and Jamnikar-Ciglenecki et al. [22]. The raw data were analysed using the Geneious 8.1.8 software. The complete genomes were obtained by mapping reads to the reference MRV genomes, obtained from GenBank (MRV Lang 1: Acc. No. M24734, AF378003, AF129820, AF461682, AF490617, AF174382, EF494445, L19774, M14325, M13139; BatMRV1-IT2011: Acc. No. KT900695 - KT900704; BDY1: Acc. No. DQ664184 - DQ664191, DQ318037, DQ312301; MRV 729: Acc. No. JN799419 - JN799428; MORV/47 Ma/06: Acc. No. KX384846 - KX384855; MRV HLJ 2007: Acc. No. HQ642769 - HQ642778; SI-MRV01: KF154724 - KF154733). When mapping to the reference was not achieved, *de-novo* assembly was performed using the Geneious 8.1.8 software with default settings. The contigs generated were then compared for similarity against all of the virus sequences deposited in the NCBI GenBank *nt* database, using BLASTn. Consensus sequences of genome segments were analysed for their ORFs, and the deduced amino-acid sequences were obtained. Phylogenetic and evolutionary analyses were conducted on all 10 MRV genome segments (ORFs) using the MEGA 6.0 software [23]. Alignment was performed with the ClustalW algorithm, followed by construction of the maximum likelihood phylogenetic tree.

### Statistical analysis

Descriptive statistics were used to characterize the study population and it’s characteristics on sampling year, gender, age, species and location of bats. For the association between virus presence in guano sample and bat’s gender, age, species as well as year and region of sample collection, a univariate logistic regression was performed separately for each of the five parameters. The level of significance for statistical tests was set to α = 0.05. Statistical analysis was carried out with the R system for statistical computing.

## Additional files

Additional file 1: Phylogenetic trees of mammalian orthoreovirus L, M and S genome segments ORFs. Black dots (●), label sequences of five Slovenian MRV isolates from bats from this study. White dot (○), label sequence of the Slovenian MRV isolate from a child with severe gastroenteritis [12]. The phylogenetic calculations were carried out using maximum likelihood, based on the Tamura-Nei model [24] and applying the best-fit models with 1000 bootstrap replicates. Bootstraps values < 50 are not shown. The scale bar represents the substitutions per site and is proportional to the genetic distance. Evolutionary analyses were conducted in MEGA 6.0 [23]. (PDF 167 kb)

Additional file 2: Details of bat guano samples included in this study. (DOCX 44 kb)

Additional file 3: Bat and sample characteristics of the pooled guano samples. (DOCX 13 kb)

## Abbreviations

DNA: Deoxyribonucleic acid
MRV: Mammalian orthoreovirus
RNA: Ribonucleic acid
RT-PCR: Reverse transcription polymerase chain reaction
