# Supplementary figures and images for: Identification of novel reassortant mammalian orthoreoviruses from bats in Slovenia
Source: BMC Vet Res. 2018 Sep 3;14:264. doi: 10.1186/s12917-018-1585-y (PMC6122641; doi:10.1186/s12917-018-1585-y)

L1

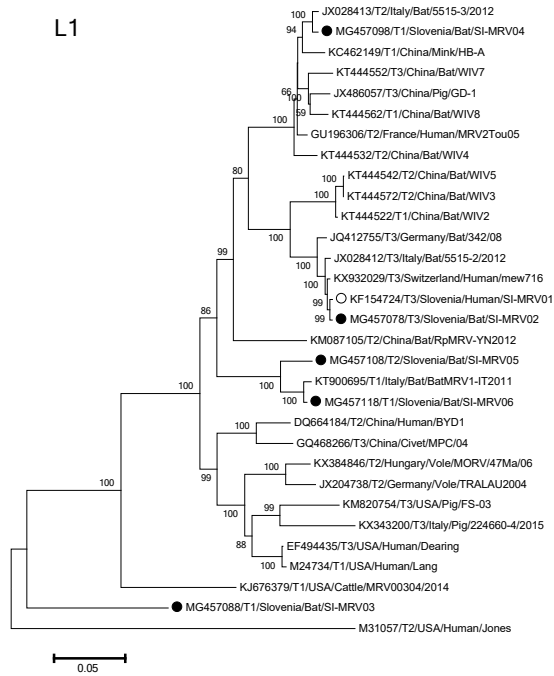

L2

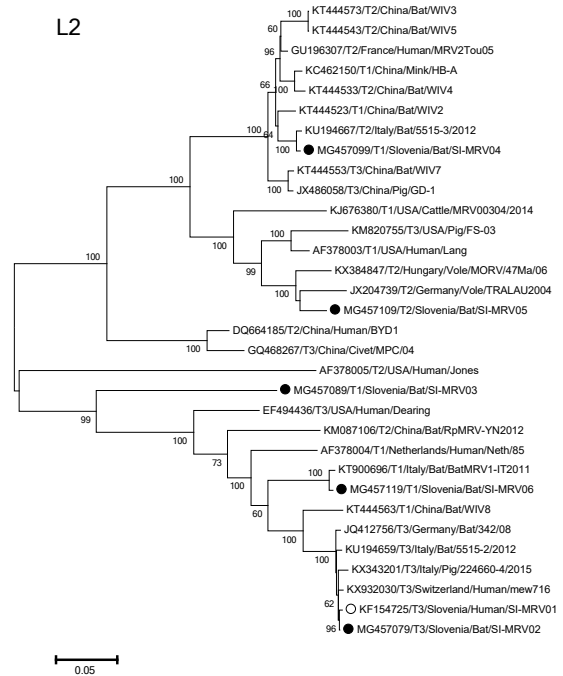

L3

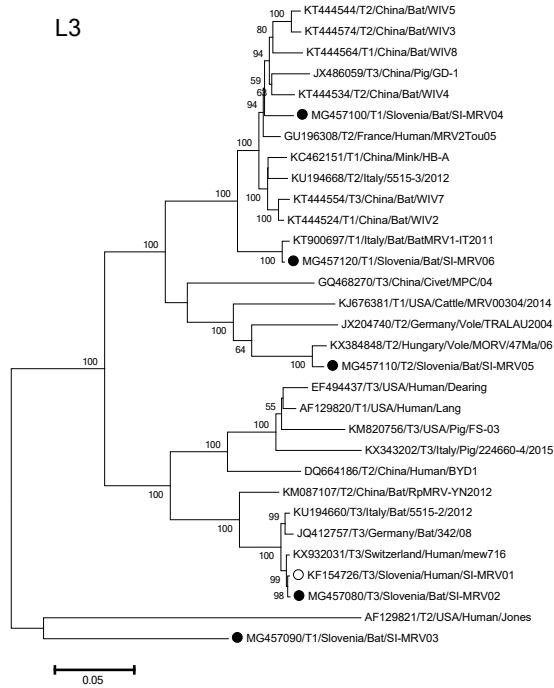

M1

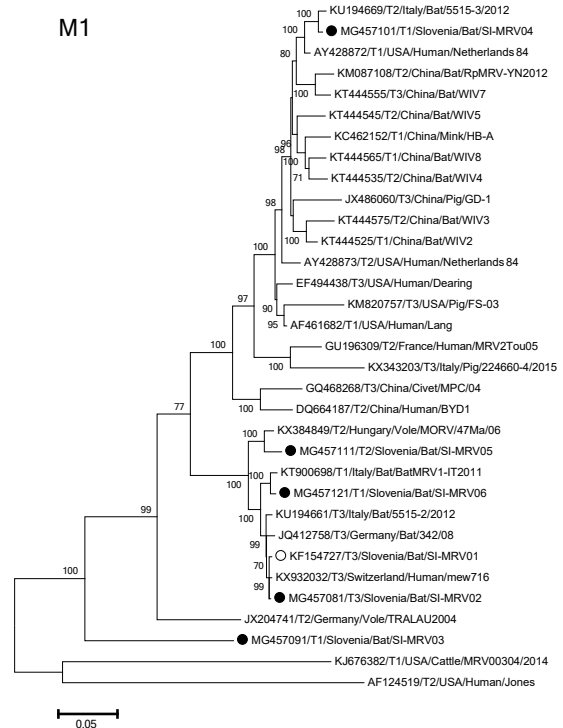

M2

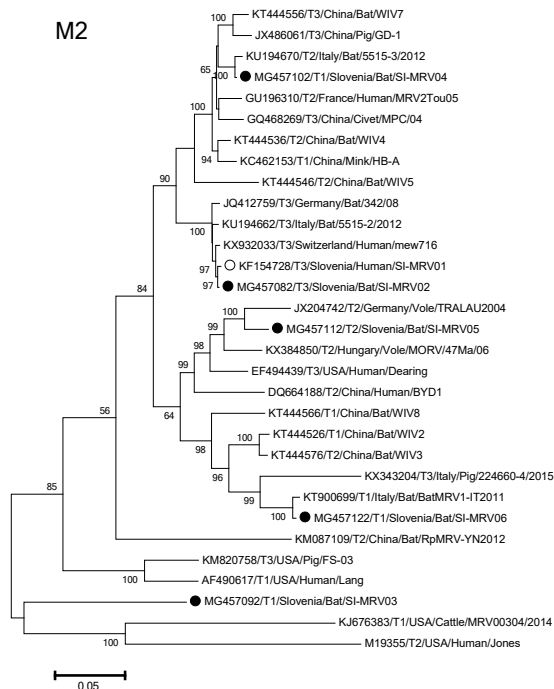

M3

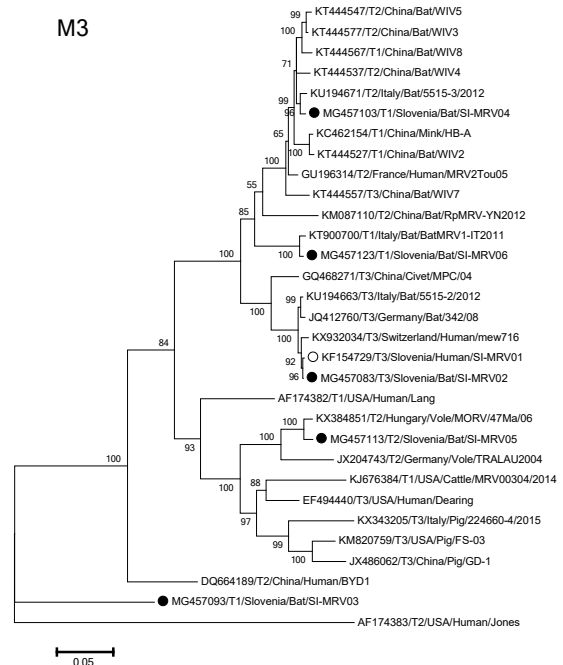

S1

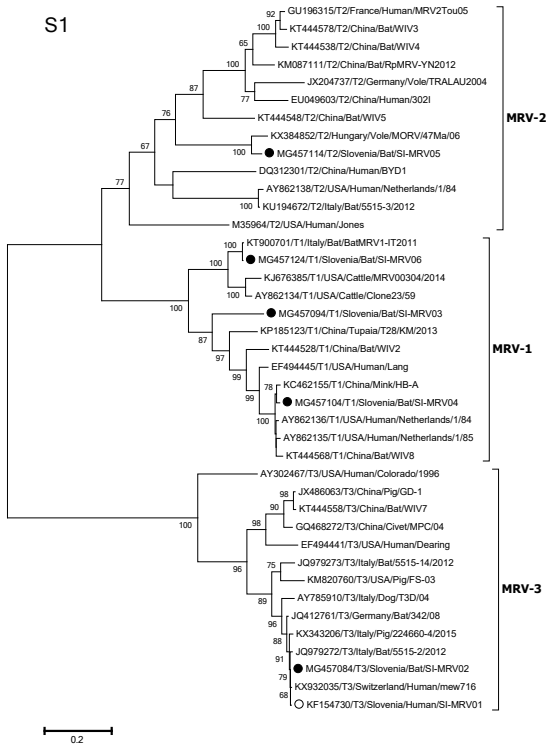

S2

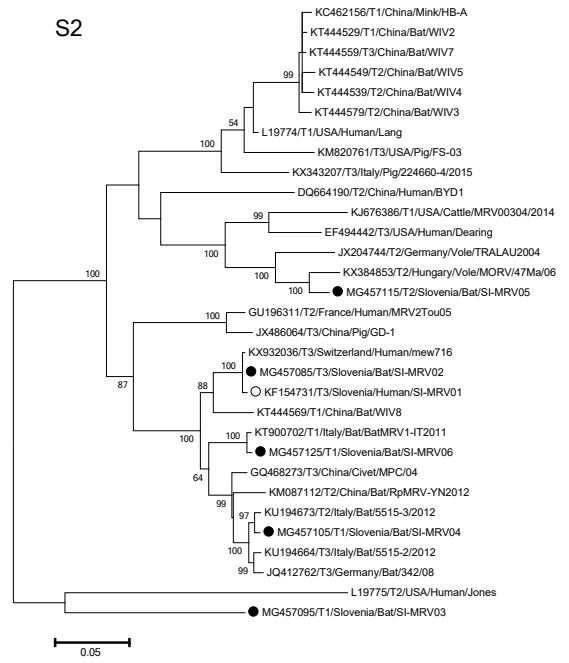

S3

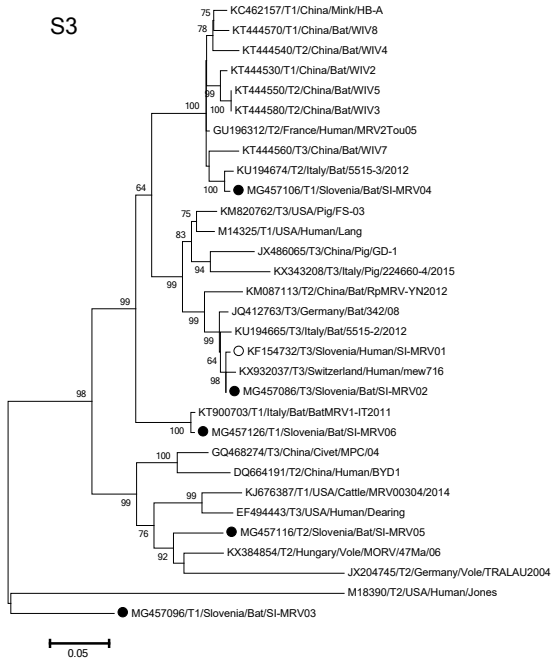

S4

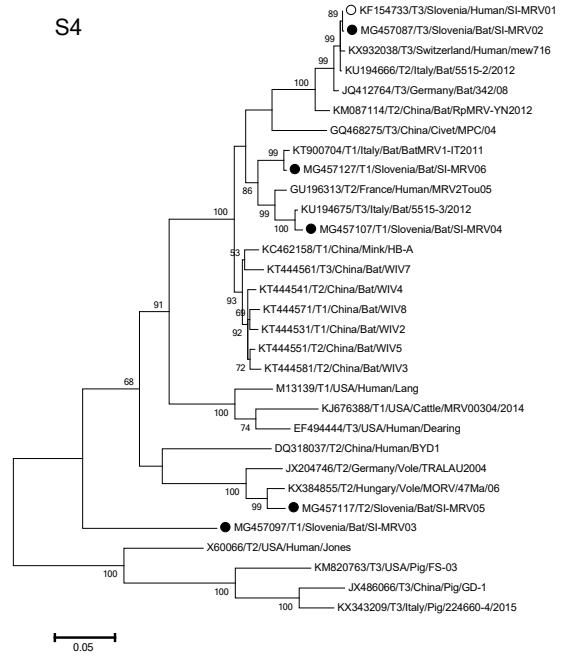

Supplement: Supplementary file 1 — Phylogenetic trees of mammalian orthoreovirus L, M and S genome segments ORFs. Black dots (●), label sequences of five Slovenian MRV isolates from bats from this study. White dot (○), label sequence of the Slovenian MRV isolate from a child with severe gastroenteritis [12]. The phylogenetic calculations were carried out using maximum likelihood, based on the Tamura-Nei model [24] and applying the best-fit models with 1000 bootstrap replicates. Bootstraps values < 50 are not shown. The scale bar represents the substitutions per site and is proportional to the genetic distance. Evolutionary analyses were conducted in MEGA 6.0 [23]. (PDF 167 kb) [file 12917_2018_1585_MOESM1_ESM.pdf]
