# Supplementary material for: Identification of novel reassortant mammalian orthoreoviruses from bats in Slovenia
Source: BMC Vet Res. 2018 Sep 3;14:264. doi: 10.1186/s12917-018-1585-y (PMC6122641; doi:10.1186/s12917-018-1585-y)
Supplement: Supplementary file 2 — Details of bat guano samples included in this study. (DOCX 44 kb) [file 12917_2018_1585_MOESM2_ESM.docx]

**Additional file 2:** Details of bat guano samples included in this study.

| **Sample code** | **Year** | **Month** | **Region** | **Bat species** | **Known status** | | **MRV RNA** |
| --- | --- | --- | --- | --- | --- | --- | --- |
|  |  |  |  |  | **Age** | **Sex** |  |
| SLO1A2357 | 2012 | unknown | Goriška | *Myotis mystacinus* | JUV | F | negative |
| SLO1A4754 | 2012 | unknown | Goriška | *Myotis blythii oxqgnathus* | AD | M | negative |
| SLO1A2356 | 2012 | unknown | Goriška | *Myotis mystacinus* | JUV | F | negative |
| SLO1A4755 | 2012 | unknown | Goriška | *Myotis blythii oxqgnathus* | AD | F | negative |
| SLO1A2358 | 2012 | unknown | Goriška | *Myotis mystacinus* | AD | M | negative |
| SLO1A4756 | 2012 | unknown | Goriška | *Myotis blythii oxqgnathus* | AD | M | negative |
| SLO1A2372 | 2012 | unknown | Pomurska | *Myotis daubentonii* | AD | F | negative |
| SLO1A4743 | 2012 | unknown | Dolenjska | *Eptesicus serotinus* | JUV | F | negative |
| SLO1A4750 | 2012 | unknown | Dolenjska | *Eptesicus serotinus* | AD | F | negative |
| SLO1A4706 | 2012 | unknown | Osrednjeslovenska | *Eptesicus serotinus* | AD | F | negative |
| SLO1A4707 | 2012 | unknown | Osrednjeslovenska | *Eptesicus serotinus* | AD | F | negative |
| SLO1A4710 | 2012 | unknown | Osrednjeslovenska | *Eptesicus serotinus* | AD | F | negative |
| SLO1A4711 | 2012 | unknown | Osrednjeslovenska | *Eptesicus serotinus* | AD | F | negative |
| SLO1A4713 | 2012 | unknown | Osrednjeslovenska | *Eptesicus serotinus* | AD | F | negative |
| SLO1A4715 | 2012 | unknown | Osrednjeslovenska | *Eptesicus serotinus* | AD | F | negative |
| SLO1A2581 | 2012 | unknown | Primorska | *Myotis daubentonii* | AD | M | negative |
| SLO1A2344 | 2012 | unknown | Osrednjeslovenska | *Myotis daubentonii* | JUV | F | negative |
| SLO1A2345 | 2012 | unknown | Osrednjeslovenska | *Myotis daubentonii* | JUV | F | negative |
| SLO1A2347 | 2012 | unknown | Osrednjeslovenska | *Myotis daubentonii* | JUV | M | negative |
| SLO1A2348 | 2012 | unknown | Osrednjeslovenska | *Myotis daubentonii* | JUV | F | negative |
| SLO1A 2349 | 2012 | unknown | Osrednjeslovenska | *Myotis daubentonii* | JUV | F | negative |
| SLO1A2373 | 2012 | unknown | Osrednjeslovenska | *Pipistrellus nathusii* | JUV | F | negative |
| SLO1A0373 | 2012 | unknown | Osrednjeslovenska | *Pipistrellus pygmaeus* | AD | M | negative |
| SLO1A2374 | 2012 | unknown | Osrednjeslovenska | *Myotis daubentonii* | JUV | M | negative |
| SLO1A2375 | 2012 | unknown | Osrednjeslovenska | *Myotis daubentonii* | AD | M | negative |
| SLO1A2376 | 2012 | unknown | Osrednjeslovenska | *Myotis daubentonii* | AD | F | negative |
| SLO1A4758 | 2012 | unknown | Osrednjeslovenska | *Nyctalus noctula* | AD | M | negative |
| SLO1A0376 | 2012 | unknown | Osrednjeslovenska | *Pipistrellus pygmaeus* | JUV | M | negative |
| SLO1A4721 | 2012 | unknown | Podravska | *Eptesicus serotinus* | AD | F | negative |
| SLO1A4469 | 2012 | unknown | Podravska | *Eptesicus serotinus* | AD | F | negative |
| SLO1A4471 | 2012 | unknown | Podravska | *Eptesicus serotinus* | AD | F | negative |
| SLO1A4724 | 2012 | unknown | Podravska | *Eptesicus serotinus* | AD | F | negative |
| SLO1A2383 | 2012 | unknown | Savinjska | *Myotis daubentonii* | AD | M | negative |
| SLO1A2384 | 2012 | unknown | Savinjska | *Myotis daubentonii* | JUV | M | negative |
| SLO1A2385 | 2012 | unknown | Savinjska | *Myotis daubentonii* | AD | M | negative |
| SLO1A2386 | 2012 | unknown | Savinjska | *Myotis daubentonii* | AD | M | negative |
| SLO1A2367 | 2012 | unknown | Osrednjeslovenska | *Myotis mystacinus* | JUV | F | negative |
| SLO1A2368 | 2012 | unknown | Dolenjska | *Myotis daubentonii* | AD | M | negative |
| SLO1A2369 | 2012 | unknown | Dolenjska | *Myotis mystacinus* | JUV | M | negative |
| SLO1A2370 | 2012 | unknown | Dolenjska | *Myotis daubentonii* | AD | M | negative |
| SLO1A2371 | 2012 | unknown | Dolenjska | *Myotis daubentonii* | AD | M | negative |
| SLO1A2294 | 2012 | unknown | Dolenjska | *Myotis daubentonii* | AD | M | negative |
| SLO1A2360 | 2012 | unknown | Dolenjska | *Myotis daubentonii* | JUV | F | negative |
| SLO1A2361 | 2012 | unknown | Dolenjska | *Myotis daubentonii* | JUV | M | positive |
| SLO1A2362 | 2012 | unknown | Dolenjska | *Myotis daubentonii* | JUV | F | negative |
| SLO1A2365 | 2012 | unknown | Dolenjska | *Myotis daubentonii* | JUV | F | positive |
| SLO1A2343 | 2012 | unknown | unknown | unknown | unknown | unknown | negative |
| SLO1A4732 | 2012 | unknown | Goriška | *Eptesicus serotinus* | AD | F | negative |
| SLO1A4450 | 2012 | unknown | Goriška | *Eptesicus serotinus* | AD | F | negative |
| SLO1A4554 | 2012 | unknown | Goriška | *Eptesicus serotinus* | AD | F | negative |
| SLO1A4243 | 2010 | Jul | Goriška | *Eptesicus serotinus* | LACTATING | F | negative |
| SLO1A4547 | 2010 | Jul | Goriška | *Eptesicus serotinus* | LACTATING | F | positive |
| SLO1A4548 | 2010 | Jul | Goriška | *Eptesicus serotinus* | LACTATING | F | negative |
| SLO1A4448 | 2010 | Jul | Goriška | *Eptesicus serotinus* | LACTATING | F | negative |
| SLO1A4549 | 2010 | Jul | Goriška | *Eptesicus serotinus* | JUV | M | negative |
| SLO1A4551 | 2010 | Jul | Goriška | *Eptesicus serotinus* | LACTATING | F | negative |
| SLO1A4558 | 2010 | Jul | Podravska | *Eptesicus serotinus* | JUV | F | negative |
| SLO1A4560 | 2010 | Jul | Podravska | *Eptesicus serotinus* | LACTATING | F | negative |
| SLO1A4562 | 2010 | Jul | Podravska | *Eptesicus serotinus* | JUV | M | negative |
| SLO1A4467 | 2010 | Jul | Podravska | *Eptesicus serotinus* | LACTATING | F | negative |
| SLO1A4259 | 2010 | Jul | Podravska | *Eptesicus serotinus* | LACTATING | F | negative |
| SLO1A4264 | 2010 | Jul | Podravska | *Eptesicus serotinus* | LACTATING | F | negative |
| SLO1A4556 | 2010 | Jul | Podravska | *Eptesicus serotinus* | JUV | F | negative |
| SLO1A4543 | 2010 | Jul | Osrednjeslovenska | *Eptesicus serotinus* | JUV | M | positive |
| SLO1A4534 | 2010 | Jul | Osrednjeslovenska | *Eptesicus serotinus* | JUV | F | positive |
| SLO1A4418 | 2010 | Jul | Osrednjeslovenska | *Eptesicus serotinus* | LACTATING | F | negative |
| SLO1A4537 | 2010 | Jul | Osrednjeslovenska | *Eptesicus serotinus* | LACTATING | F | negative |
| SLO1A4412 | 2010 | Jul | Osrednjeslovenska | *Eptesicus serotinus* | LACTATING | F | positive |
| SLO1A4542 | 2010 | Jul | Osrednjeslovenska | *Eptesicus serotinus* | JUV | F | positive |
| SLO1A4541 | 2010 | Jul | Osrednjeslovenska | *Eptesicus serotinus* | JUV | F | positive |
| SLO1A4535 | 2010 | Jul | Osrednjeslovenska | *Eptesicus serotinus* | LACTATING | F | negative |
| SLO1A4532 | 2010 | Jul | Osrednjeslovenska | *Eptesicus serotinus* | LACTATING | F | positive |
| SLO1A4528 | 2010 | Jul | Osrednjeslovenska | *Eptesicus serotinus* | LACTATING | F | negative |
| SLO1A4527 | 2010 | Jul | Osrednjeslovenska | *Eptesicus serotinus* | JUV | F | negative |
| SLO1A4531 | 2010 | Jul | Osrednjeslovenska | *Eptesicus serotinus* | AD | F | negative |
| SLO1A4529 | 2010 | Jul | Osrednjeslovenska | *Eptesicus serotinus* | AD | F | positive |
| SLO1A4234 | 2010 | Jul | Osrednjeslovenska | *Eptesicus serotinus* | LACTATING | F | positive |
| SLO1A4569 | 2010 | Jul | Osrednjeslovenska | *Eptesicus serotinus* | LACTATING | F | positive |
| SLO1A4573 | 2010 | Jul | Osrednjeslovenska | *Eptesicus serotinus* | LACTATING | F | positive |
| SLO1A4568 | 2010 | Jul | Osrednjeslovenska | *Eptesicus serotinus* | LACTATING | F | positive |
| SLO1A4563 | 2010 | Jul | Osrednjeslovenska | *Eptesicus serotinus* | LACTATING | F | negative |
| SLO1A4565 | 2010 | Jul | Osrednjeslovenska | *Eptesicus serotinus* | JUV | F | positive |
| SLO1A4566 | 2010 | Jul | Osrednjeslovenska | *Eptesicus serotinus* | LACTATING | F | positive |
| SLO1A4571 | 2010 | Jul | Osrednjeslovenska | *Eptesicus serotinus* | JUV | F | positive |
| CKFF03 | 2010 | Aug | Savinjska | *Miniopterus schreibersii* | AD | M | positive |
| SLO1A2282 | 2010 | Aug | Savinjska | *Myotis daubentonii* | AD | M | positive |
| CKFF04 | 2010 | Aug | Savinjska | *Miniopterus schreibersii* | AD | M | positive |
| CKFF05 | 2010 | Aug | Savinjska | *Miniopterus schreibersii* | AD | M | positive |
| SLO1A2285 | 2010 | Aug | Primorska | *Myotis daubentonii* | AD | M | negative |
| CKFF35 | 2010 | Aug | Primorska | *Myotis oxygnathus* | LACTATING | F | negative |
| CKFF40 | 2010 | Aug | Primorska | *Myotis oxygnathus* | AD | M | negative |
| CKFF30 | 2010 | Aug | Primorska | *Miniopterus schreibersii* | JUV | F | negative |
| CKFF32 | 2010 | Aug | Primorska | *Miniopterus schreibersii* | JUV | F | negative |
| CKFF33 | 2010 | Aug | Primorska | *Miniopterus schreibersii* | JUV | F | negative |
| CKFF44 | 2010 | Aug | Primorska | *Miniopterus schreibersii* | JUV | M | negative |
| CKFF45 | 2010 | Aug | Primorska | *Miniopterus schreibersii* | JUV | F | negative |
| CKFF46 | 2010 | Aug | Primorska | *Miniopterus schreibersii* | JUV | F | negative |
| SLO1A4610 | 2010 | Aug | Dolenjska | *Myotis myotis* | LACTATING | F | negative |
| SLO1A0169 | 2010 | Aug | Dolenjska | *Myotis mystacinus* | JUV | F | negative |
| SLO1A2287 | 2010 | Aug | Dolenjska | *Rhinolophus hipposideros* | AD | M | positive |
| SLO1A2289 | 2010 | Aug | Dolenjska | *Myotis daubentonii* | AD | M | positive |
| CKFF16 | 2010 | Aug | Podravska | *Miniopterus schreibersii* | AD | M | negative |
| CKFF07 | 2010 | Aug | Podravska | *Miniopterus schreibersii* | LACTATING | F | negative |
| SLO1A2014 | 2010 | Aug | Podravska | *Miniopterus schreibersii* | AD | M | positive |
| CKFF13 | 2010 | Aug | Podravska | *Miniopterus schreibersii* | LACTATING | F | positive |
| CKFF10 | 2010 | Aug | Podravska | *Miniopterus schreibersii* | AD | M | negative |
| SLO1A4578 | 2010 | Aug | Podravska | *Myotis myotis* | LACTATING | F | negative |
| CKFF06 | 2010 | Aug | Podravska | *Miniopterus schreibersii* | LACTATING | F | positive |
| SLO1A2061 | 2010 | Aug | Podravska | *Miniopterus schreibersii* | LACTATING | F | positive |
| SLO1A4577 | 2010 | Aug | Podravska | *Myotis myotis* | LACTATING | F | positive |
| SLO1A4582 | 2010 | Aug | Podravska | *Myotis myotis* | LACTATING | F | negative |
| CKFF11 | 2010 | Aug | Podravska | *Miniopterus schreibersii* | LACTATING | F | positive |
| SLO1A4575 | 2010 | Aug | Podravska | *Myotis myotis* | LACTATING | F | positive |
| SLO1A4576 | 2010 | Aug | Podravska | *Myotis myotis* | AD | M | positive |
| SLO1A4580 | 2010 | Aug | Podravska | *Myotis myotis* | AD | M | positive |
| SLO1A4583 | 2010 | Aug | Podravska | *Myotis myotis* | AD | M | negative |
| SLO1A4544 | 2010 | Aug | Goriška | *Eptesicus serotinus* | LACTATING | F | negative |
| SLO1A4536 | 2010 | Aug | Podravska | *Eptesicus serotinus* | LACTATING | F | negative |
| SLO1A4533 | 2010 | Aug | Osrednjeslovenska | *Eptesicus serotinus* | LACTATING | F | negative |
| SLO1A4604 | 2010 | Aug | Dolenjska | *Eptesicus serotinus* | JUV | F | positive |
| SLO1A4605 | 2010 | Aug | Dolenjska | *Eptesicus serotinus* | JUV | F | negative |
| SLO1A4606 | 2010 | Aug | Dolenjska | *Eptesicus serotinus* | LACTATING | F | negative |
| SLO1A4607 | 2010 | Aug | Dolenjska | *Eptesicus serotinus* | JUV | F | negative |
| SLO1A4237 | 2010 | Aug | Dolenjska | *Eptesicus serotinus* | LACTATING | F | positive |
| SLO1A0170 | 2010 | Aug | Primorska | *Pipistrellus pygmaeus* | AD | M | negative |
| SLO1A0171 | 2010 | Aug | Primorska | *Myotis mystacinus* | AD | M | negative |
| SLO1A2299 | 2010 | Aug | Primorska | *Myotis mystacinus* | JUV | F | negative |
| SLO1A2300 | 2010 | Aug | Primorska | *Myotis daubentonii* | AD | M | negative |
| CKFF47 | 2010 | Aug | Primorska | *Myotis daubentonii* | AD | M | negative |
| CKFF49 | 2010 | Aug | Primorska | *Myotis mystacinus* | LACTATING | F | negative |
| SLO1A2309 | 2010 | Sep | Podravska | *Myotis daubentonii* | JUV | M | negative |
| SLO1A4615 | 2010 | Sep | Savinjska | *Myotis myotis* | AD | M | negative |
| SLO1A2310 | 2010 | Sep | Savinjska | *Myotis daubentonii* | AD | M | negative |
| SLO1A4616 | 2010 | Sep | Savinjska | *Myotis myotis* | JUV | F | negative |
| SLO1A4611 | 2010 | Sep | Savinjska | *Myotis myotis* | AD | M | negative |
| SLO1A4612 | 2010 | Sep | Savinjska | *Myotis myotis* | AD | M | negative |
| SLO1A4613 | 2010 | Sep | Savinjska | *Myotis myotis* | AD | M | negative |
| SLO1A4614 | 2010 | Sep | Savinjska | *Myotis myotis* | JUV | F | negative |
| SLO1A2305 | 2010 | Sep | Savinjska | *Myotis daubentonii* | AD | M | negative |
| SLO1A2306 | 2010 | Sep | Savinjska | *Myotis daubentonii* | AD | M | negative |
| SLO1A0150 | 2010 | Sep | Osrednjeslovenska | *Pipistrellus pygmaeus* | JUV | F | negative |
| SLO1A2301 | 2010 | Sep | Osrednjeslovenska | *Myotis daubentonii* | AD | F | negative |
| SLO1A2302 | 2010 | Sep | Osrednjeslovenska | *Myotis daubentonii* | AD | M | negative |
| SLO1A2303 | 2010 | Sep | Osrednjeslovenska | *Myotis daubentonii* | JUV | F | negative |
| SLO1A2304 | 2010 | Sep | Osrednjeslovenska | *Myotis daubentonii* | JUV | F | negative |
| SLO1A2268 | 2010 | unknown | unknown | unknown | unknown | unknown | negative |
| SLO1A2881 | 2010 | unknown | unknown | unknown | unknown | unknown | positive |
| SLO1A4221 | 2009 | May | Osrednjeslovenska | *Eptesicus serotinus* | LACTATING | F | negative |
| SLO1A4412 | 2009 | May | Osrednjeslovenska | *Eptesicus serotinus* | LACTATING | F | negative |
| SLO1A4413 | 2009 | May | Osrednjeslovenska | *Eptesicus serotinus* | LACTATING | F | negative |
| SLO1A4414 | 2009 | May | Osrednjeslovenska | *Eptesicus serotinus* | LACTATING | F | negative |
| SLO1A4415 | 2009 | May | Osrednjeslovenska | *Eptesicus serotinus* | LACTATING | F | negative |
| SLO1A4416 | 2009 | May | Osrednjeslovenska | *Eptesicus serotinus* | LACTATING | F | negative |
| SLO1A4417 | 2009 | May | Osrednjeslovenska | *Eptesicus serotinus* | LACTATING | F | negative |
| SLO1A4418 | 2009 | May | Osrednjeslovenska | *Eptesicus serotinus* | LACTATING | F | positive |
| SLO1A 4443 | 2009 | May | Podravska | *Eptesicus serotinus* | GRAVID | F | negative |
| SLO1A2230 | 2009 | May | Dolenjska | *Myotis emarginatus* | LACTATING | F | negative |
| SLO1A2231 | 2009 | May | Dolenjska | *Myotis emarginatus* | LACTATING | F | negative |
| SLO1A2233 | 2009 | May | Dolenjska | *Myotis emarginatus* | LACTATING | F | negative |
| SLO1A4429 | 2009 | May | Osrednjeslovenska | *Eptesicus serotinus* | LACTATING | F | negative |
| SLO1A 4430 | 2009 | May | Osrednjeslovenska | *Eptesicus serotinus* | LACTATING | F | negative |
| SLO1A2224 | 2009 | May | Dolenjska | *Myotis emarginatus* | JUV | F | negative |
| SLO1A2225 | 2009 | May | Dolenjska | *Myotis emarginatus* | LACTATING | F | negative |
| SLO1A2226 | 2009 | May | Dolenjska | *Myotis emarginatus* | LACTATING | F | negative |
| SLO1A2227 | 2009 | May | Dolenjska | *Myotis emarginatus* | LACTATING | F | negative |
| SLO1A2228 | 2009 | May | Dolenjska | *Myotis emarginatus* | LACTATING | F | negative |
| SLO1A 2229 | 2009 | May | Dolenjska | *Myotis emarginatus* | LACTATING | F | negative |
| SLO1A4439 | 2009 | May | Dolenjska | *Eptesicus serotinus* | LACTATING | F | negative |
| SLO1A4440 | 2009 | May | Dolenjska | *Eptesicus serotinus* | LACTATING | F | negative |
| SLO1A4441 | 2009 | May | Dolenjska | *Eptesicus serotinus* | LACTATING | F | negative |
| SLO1A0117 | 2009 | May | Goriška | *Myotis mystacinus* | GRAVID | F | negative |
| SLO1A0118 | 2009 | May | Goriška | *Myotis mystacinus* | GRAVID | F | negative |
| SLO1A4422 | 2009 | May | Osrednjeslovenska | *Eptesicus serotinus* | LACTATING | F | negative |
| SLO1A 2213 | 2009 | May | Podravska | *Myotis emarginatus* | LACTATING | F | negative |
| SLO1A2214 | 2009 | May | Podravska | *Myotis emarginatus* | LACTATING | F | negative |
| SLO1A2215 | 2009 | May | Podravska | *Myotis emarginatus* | LACTATING | F | negative |
| SLO1A2216 | 2009 | May | Podravska | *Myotis emarginatus* | LACTATING | F | negative |
| SLO1A2217 | 2009 | May | Podravska | *Myotis emarginatus* | AD | F | negative |
| SLO1A2218 | 2009 | May | Podravska | *Myotis emarginatus* | AD | F | negative |
| SLO1A4423 | 2009 | May | Dolenjska | *Eptesicus serotinus* | LACTATING | F | negative |
| SLO1A4425 | 2009 | May | Dolenjska | *Eptesicus serotinus* | LACTATING | F | negative |
| SLO1A4426 | 2009 | May | Dolenjska | *Eptesicus serotinus* | LACTATING | F | negative |
| SLO1A4428 | 2009 | May | Dolenjska | *Eptesicus serotinus* | LACTATING | F | negative |
| SLO1A4427 | 2009 | May | Dolenjska | *Eptesicus serotinus* | LACTATING | F | negative |
| SLO1A4433 | 2009 | May | Osrednjeslovenska | *Eptesicus serotinus* | LACTATING | F | negative |
| SLO1A2191 | 2009 | May | Osrednjeslovenska | *Myotis emarginatus* | LACTATING | F | negative |
| SLO1A2192 | 2009 | May | Osrednjeslovenska | *Myotis emarginatus* | LACTATING | F | negative |
| SLO1A2194 | 2009 | May | Osrednjeslovenska | *Myotis emarginatus* | LACTATING | F | negative |
| SLO1A2195 | 2009 | May | Osrednjeslovenska | *Myotis emarginatus* | LACTATING | F | negative |
| SLO1A2196 | 2009 | May | Osrednjeslovenska | *Myotis emarginatus* | LACTATING | F | negative |
| SLO1A2197 | 2009 | May | Osrednjeslovenska | *Myotis emarginatus* | LACTATING | F | negative |
| SLO1A2198 | 2009 | May | Osrednjeslovenska | *Myotis emarginatus* | LACTATING | F | positive |
| SLO1A2200 | 2009 | May | Osrednjeslovenska | *Myotis emarginatus* | LACTATING | F | positive |
| SLO1A2199 | 2009 | May | Osrednjeslovenska | *Myotis emarginatus* | LACTATING | F | positive |
| SLO1A2201 | 2009 | May | Osrednjeslovenska | *Myotis emarginatus* | LACTATING | F | negative |
| SLO1A2203 | 2009 | May | Gorenjska | *Myotis emarginatus* | LACTATING | F | negative |
| SLO1A2205 | 2009 | May | Gorenjska | *Myotis emarginatus* | JUV | F | negative |
| SLO1A2207 | 2009 | May | Gorenjska | *Myotis emarginatus* | JUV | F | negative |
| SLO1A2204 | 2009 | May | Gorenjska | *Myotis emarginatus* | LACTATING | F | negative |
| SLO1A0109 | 2009 | May | Pomurska | *Pipistrellus kuhlii* | GRAVID | F | negative |
| SLO1A0110 | 2009 | May | Pomurska | *Pipistrellus kuhlii* | GRAVID | F | negative |
| SLO1A0111 | 2009 | May | Pomurska | *Pipistrellus kuhlii* | GRAVID | F | negative |
| SLO1A0112 | 2009 | May | Pomurska | *Pipistrellus kuhlii* | AD | M | negative |
| SLO1A0113 | 2009 | May | Pomurska | *Pipistrellus kuhlii* | GRAVID | F | negative |
| SLO1A0115 | 2009 | May | Pomurska | *Pipistrellus kuhlii* | GRAVID | F | negative |
| SLO1A0041 | 2009 | May | Osrednjeslovenska | *Pipistrellus kuhlii* | JUV | F | negative |
| SLO1A0119 | 2009 | May | Osrednjeslovenska | *Pipistrellus kuhlii* | GRAVID | F | negative |
| SLO1A2235 | 2009 | May | Gorenjska | *Myotis emarginatus* | JUV | F | negative |
| SLO1A2237 | 2009 | May | Gorenjska | *Myotis emarginatus* | LACTATING | F | negative |
| SLO1A2238 | 2009 | May | Gorenjska | *Myotis emarginatus* | LACTATING | F | negative |
| SLO1A2212 | 2009 | May | Goriška | *Myotis emarginatus* | LACTATING | F | negative |
| SLO1A4244 | 2009 | Jul | Goriška | *Eptesicus serotinus* | LACTATING | F | negative |
| SLO1A4446 | 2009 | Jul | Goriška | *Eptesicus serotinus* | LACTATING | F | negative |
| SLO1A4447 | 2009 | Jul | Goriška | *Eptesicus serotinus* | LACTATING | F | negative |
| SLO1A4448 | 2009 | Jul | Goriška | *Eptesicus serotinus* | LACTATING | F | negative |
| SLO1A4449 | 2009 | Jul | Goriška | *Eptesicus serotinus* | JUV | F | negative |
| SLO1A4451 | 2009 | Jul | Goriška | *Eptesicus serotinus* | LACTATING | F | negative |
| SLO1A4452 | 2009 | Jul | Goriška | *Eptesicus serotinus* | LACTATING | F | negative |
| SLO1A4453 | 2009 | Jul | Goriška | *Eptesicus serotinus* | LACTATING | F | negative |
| SLO1A4455 | 2009 | Jul | Goriška | *Eptesicus serotinus* | LACTATING | F | negative |
| SLO1A4449 | 2009 | Jul | Goriška | *Eptesicus serotinus* | JUV | F | positive |
| SLO1A4467 | 2009 | Jul | Podravska | *Eptesicus serotinus* | LACTATING | F | negative |
| SLO1A4468 | 2009 | Jul | Podravska | *Eptesicus serotinus* | LACTATING | F | negative |
| SLO1A4469 | 2009 | Jul | Podravska | *Eptesicus serotinus* | LACTATING | F | negative |
| SLO1A4470 | 2009 | Jul | Podravska | *Eptesicus serotinus* | LACTATING | F | negative |
| SLO1A4471 | 2009 | Jul | Podravska | *Eptesicus serotinus* | JUV | F | negative |
| SLO1A4475 | 2009 | Jul | Podravska | *Eptesicus serotinus* | JUV | M | negative |
| SLO1A4476 | 2009 | Jul | Podravska | *Eptesicus serotinus* | JUV | F | negative |
| SLO1A4481 | 2009 | Jul | Osrednjeslovenska | *Eptesicus serotinus* | LACTATING | F | negative |
| SLO1A4483 | 2009 | Jul | Osrednjeslovenska | *Eptesicus serotinus* | LACTATING | F | negative |
| SLO1A4485 | 2009 | Jul | Osrednjeslovenska | *Eptesicus serotinus* | LACTATING | F | negative |
| SLO1A4486 | 2009 | Jul | Osrednjeslovenska | *Eptesicus serotinus* | JUV | M | negative |
| SLO1A4487 | 2009 | Jul | Osrednjeslovenska | *Eptesicus serotinus* | JUV | F | negative |
| SLO1A4456 | 2009 | Jul | Goriška | *Eptesicus serotinus* | LACTATING | F | negative |
| SLO1A4458 | 2009 | Jul | Goriška | *Eptesicus serotinus* | LACTATING | F | negative |
| SLO1A4459 | 2009 | Jul | Goriška | *Eptesicus serotinus* | LACTATING | F | negative |
| SLO1A4460 | 2009 | Jul | Goriška | *Eptesicus serotinus* | JUV | F | negative |
| SLO1A4462 | 2009 | Jul | Goriška | *Eptesicus serotinus* | LACTATING | F | negative |
| SLO1A4463 | 2009 | Jul | Goriška | *Eptesicus serotinus* | LACTATING | F | negative |
| SLO1A2240 | 2009 | Jul | Dolenjska | *Myotis daubentonii* | AD | M | negative |
| SLO1A2241 | 2009 | Jul | Dolenjska | *Myotis daubentonii* | AD | M | negative |
| SLO1A2242 | 2009 | Jul | Dolenjska | *Myotis daubentonii* | AD | M | negative |
| SLO1A2246 | 2009 | Aug | Gorenjska | *Myotis daubentonii* | AD | M | negative |
| SLO1A4493 | 2009 | Aug | Osrednjeslovenska | *Nyctalus noctula* | JUV | F | negative |
| SLO1A2245 | 2009 | Aug | Primorska | *Nyctalus leisleri* | AD | M | negative |
| SLO1A2243 | 2009 | Aug | Primorska | *Nyctalus leisleri* | AD | M | negative |
| SLO1A4492 | 2009 | Aug | Primorska | *Nyctalus noctula* | JUV | F | negative |
| SLO1A4491 | 2009 | Aug | Primorska | *Nyctalus leisleri* | JUV | M | negative |
| SLO1A2248 | 2009 | Aug | Primorska | *Myotis mystacinus s.lat.* | LACTATING | F | negative |
| SLO1A2247 | 2009 | Aug | Primorska | *Myotis daubentonii* | AD | M | negative |
| SLO1A2261 | 2009 | Sep | Dolenjska | *Myotis bechsteinii* | AD | M | negative |
| SLO1A2250 | 2009 | Sep | Primorska | *Myotis daubentonii* | AD | M | negative |
| SLO1A4496 | 2009 | Sep | Primorska | *Nyctalus noctula* | JUV | F | negative |
| SLO1A4497 | 2009 | Sep | Primorska | *Nyctalus noctula* | JUV | F | negative |
| SLO1A2249 | 2009 | Sep | Savinjska | *Myotis daubentonii* | AD | M | negative |
| SLO1A4494 | 2009 | Sep | Savinjska | *Eptesicus serotinus* | AD | M | negative |
| SLO1A4495 | 2009 | Sep | Savinjska | *Eptesicus serotinus* | AD | M | negative |
| SLO1A2260 | 2009 | Sep | Osrednjeslovenska | *Myotis daubentonii* | JUV | F | negative |
| SLO1A2252 | 2009 | Sep | Gorenjska | *Myotis daubentonii* | AD | M | negative |
| SLO1A2255 | 2009 | Sep | Gorenjska | *Myotis mystacinus* | AD | M | negative |
| SLO1A2257 | 2009 | Sep | Gorenjska | *Myotis daubentonii* | AD | M | negative |
| SLO1A2256 | 2009 | Sep | Gorenjska | *Myotis mystacinus* | AD | M | negative |
| SLO1A4501 | 2009 | Sep | Gorenjska | *Nyctalus noctula* | AD | M | negative |
| SLO1A4502 | 2009 | Sep | Gorenjska | *Nyctalus noctula* | AD | F | negative |
| SLO1A2262 | 2009 | Sep | Primorska | *Myotis daubentonii* | AD | M | negative |
| SLO1A2263 | 2009 | Sep | Primorska | *Myotis daubentonii* | AD | M | negative |
| SLO1A2264 | 2009 | Sep | Primorska | *Myotis daubentonii* | AD | M | negative |
| SLO1A2265 | 2009 | Sep | Primorska | *Myotis daubentonii* | AD | M | negative |
| SLO1A2266 | 2009 | Sep | Primorska | *Myotis daubentonii* | AD | M | negative |
| SLO1A2267 | 2009 | Sep | Primorska | *Myotis daubentonii* | AD | M | negative |
| SLO1A2268 | 2009 | Sep | Primorska | *Myotis daubentonii* | JUV | M | negative |
| SLO1A2269 | 2009 | Sep | Primorska | *Myotis daubentonii* | AD | M | negative |
| SLO1A2270 | 2009 | Sep | Primorska | *Myotis daubentonii* | AD | M | negative |
| SLO1A2272 | 2009 | Sep | Primorska | *Myotis daubentonii* | AD | M | negative |
| SLO1A2273 | 2009 | Sep | Primorska | *Myotis daubentonii* | AD | M | positive |
| SLO1A2251 | 2009 | Sep | Osrednjeslovenska | *Myotis daubentonii* | JUV | M | negative |
| SLO1A4499 | 2009 | Sep | Osrednjeslovenska | *Nyctalus noctula* | AD | M | negative |
| SLO1A4500 | 2009 | Sep | Osrednjeslovenska | *Nyctalus noctula* | LACTATING | F | negative |
| SLO1A4503 | 2009 | Sep | Dolenjska | *Myotis myotis* | LACTATING | F | negative |
| CKFF107 | 2009 | Sep | Dolenjska | *Miniopterus schreibersii* | AD | M | negative |
| SLO1A0140 | 2009 | Sep | Osrednjeslovenska | *Pipistrellus pygmaeus* | LACTATING | F | negative |
| SLO1A0142 | 2009 | Sep | Osrednjeslovenska | *Pipistrellus pygmaeus* | AD | M | negative |
| SLO1A0143 | 2009 | Sep | Osrednjeslovenska | *Pipistrellus pygmaeus* | JUV | F | negative |
| SLO1A0144 | 2009 | Sep | Osrednjeslovenska | *Pipistrellus pygmaeus* | JUV | F | negative |
| SLO1A0147 | 2009 | Sep | Goriška | *Pipistrellus pygmaeus* | AD | M | negative |
| SLO1A0145 | 2009 | Sep | Osrednjeslovenska | *Pipistrellus pygmaeus* | AD | M | negative |
| SLO1A0146 | 2009 | Sep | Osrednjeslovenska | *Pipistrellus pipistrellus* | LACTATING | F | negative |
| SLO1A2161 | 2009 | Sep | Dolenjska | *Myotis daubentonii* | AD | M | negative |
| SLO1A2275 | 2009 | Oct | Osrednjeslovenska | *Myotis daubentonii* | AD | M | negative |
| SLO1A2274 | 2009 | Oct | Osrednjeslovenska | *Myotis daubentonii* | AD | M | negative |
| SLO1A2136 | 2009 | unknown | unknown | unknown | unknown | unknown | negative |
| SLO1A4200 | 2009 | unknown | unknown | unknown | unknown | unknown | negative |
| SLO1A4206 | 2009 | unknown | unknown | unknown | unknown | unknown | negative |
| SLO1A4215 | 2009 | unknown | unknown | unknown | unknown | unknown | negative |
| SLO1A4231 | 2009 | unknown | unknown | unknown | unknown | unknown | negative |
| SLO1A2137 | 2009 | unknown | unknown | unknown | unknown | unknown | negative |
| SLO1A2138 | 2009 | unknown | unknown | unknown | unknown | unknown | negative |
| SLO1A2139 | 2009 | unknown | unknown | unknown | unknown | unknown | negative |
| SLO1A2140 | 2009 | unknown | unknown | unknown | unknown | unknown | negative |
| SLO1A2143 | 2009 | unknown | unknown | unknown | unknown | unknown | negative |
| SLO1A2142 | 2009 | unknown | unknown | unknown | unknown | unknown | negative |
| SLO1A4199 | 2009 | unknown | unknown | unknown | unknown | unknown | negative |
| SLO1A4202 | 2009 | unknown | unknown | unknown | unknown | unknown | negative |
| SLO1A4224 | 2009 | unknown | unknown | unknown | unknown | unknown | negative |
| SLO1A4225 | 2009 | unknown | unknown | unknown | unknown | unknown | negative |
| SLO1A4228 | 2009 | unknown | unknown | unknown | unknown | unknown | negative |
| SLO1A4232 | 2009 | unknown | unknown | unknown | unknown | unknown | negative |
| SLO1A4234 | 2009 | unknown | unknown | unknown | unknown | unknown | negative |
| SLO1A4235 | 2009 | unknown | unknown | unknown | unknown | unknown | negative |
| SLO1A4239 | 2009 | unknown | unknown | unknown | unknown | unknown | negative |
| SLO1A4242 | 2009 | unknown | unknown | unknown | unknown | unknown | negative |
| SLO1A4243 | 2009 | unknown | unknown | unknown | unknown | unknown | negative |
| SLO1A4246 | 2009 | unknown | unknown | unknown | unknown | unknown | negative |
| SLO1A4247 | 2009 | unknown | unknown | unknown | unknown | unknown | negative |
| SLO1A4249 | 2009 | unknown | unknown | unknown | unknown | unknown | negative |
| SLO1A4251 | 2009 | unknown | unknown | unknown | unknown | unknown | negative |
| SLO1A4252 | 2009 | unknown | unknown | unknown | unknown | unknown | negative |
| SLO1A4254 | 2009 | unknown | unknown | unknown | unknown | unknown | negative |
| SLO1A4255 | 2009 | unknown | unknown | unknown | unknown | unknown | negative |
| SLO1A4256 | 2009 | unknown | unknown | unknown | unknown | unknown | negative |
| SLO1A4257 | 2009 | unknown | unknown | unknown | unknown | unknown | negative |
| SLO1A4258 | 2009 | unknown | unknown | unknown | unknown | unknown | negative |
| SLO1A4261 | 2009 | unknown | unknown | unknown | unknown | unknown | negative |
| SLO1A4262 | 2009 | unknown | unknown | unknown | unknown | unknown | positive |
| SLO1A4358 | 2009 | unknown | unknown | unknown | unknown | unknown | negative |
| SLO1A4359 | 2009 | unknown | unknown | unknown | unknown | unknown | negative |
| SLO1A4360 | 2009 | unknown | unknown | unknown | unknown | unknown | negative |
| SLO1A4401 | 2009 | unknown | unknown | unknown | unknown | unknown | negative |
| SLO1A4408 | 2009 | unknown | unknown | unknown | unknown | unknown | negative |
| SLO1A4421 | 2009 | unknown | unknown | unknown | unknown | unknown | negative |
| CKFF13 | 2009 | unknown | unknown | unknown | unknown | unknown | negative |
| CKFF14 | 2009 | unknown | unknown | unknown | unknown | unknown | negative |
| CKFF15 | 2009 | unknown | unknown | unknown | unknown | unknown | negative |
| SLO1A4245 | 2008 | May | Goriška | *Eptesicus serotinus* | GRAVID | F | negative |
| SLO1A4212 | 2008 | May | Osrednjeslovenska | *Eptesicus serotinus* | AD | F | negative |
| SLO1A4213 | 2008 | May | Osrednjeslovenska | *Eptesicus serotinus* | AD | F | negative |
| SLO1A4214 | 2008 | May | Osrednjeslovenska | *Eptesicus serotinus* | AD | F | negative |
| SLO1A4216 | 2008 | May | Osrednjeslovenska | *Eptesicus serotinus* | AD | F | negative |
| SLO1A4217 | 2008 | May | Osrednjeslovenska | *Eptesicus serotinus* | GRAVID | F | negative |
| SLO1A4218 | 2008 | May | Osrednjeslovenska | *Eptesicus serotinus* | GRAVID | F | negative |
| SLO1A4221 | 2008 | May | Osrednjeslovenska | *Eptesicus serotinus* | AD | F | negative |
| SLO1A4222 | 2008 | May | Osrednjeslovenska | *Eptesicus serotinus* | AD | F | negative |
| SLO1A4212 | 2008 | May | Osrednjeslovenska | *Eptesicus serotinus* | AD | F | negative |
| SLO1A2133 | 2008 | May | Osrednjeslovenska | *Rhinolophus hipposideros* | GRAVID | F | negative |
| SLO1A2134 | 2008 | May | Osrednjeslovenska | *Rhinolophus hipposideros* | GRAVID | F | negative |
| SLO1A2135 | 2008 | May | Osrednjeslovenska | *Rhinolophus hipposideros* | GRAVID | F | negative |
| SLO1A4203 | 2008 | May | Osrednjeslovenska | *Eptesicus serotinus* | AD | F | negative |
| SLO1A4204 | 2008 | May | Osrednjeslovenska | *Eptesicus serotinus* | AD | F | negative |
| SLO1A4205 | 2008 | May | Osrednjeslovenska | *Eptesicus serotinus* | AD | F | negative |
| SLO1A4209 | 2008 | May | Osrednjeslovenska | *Myotis myotis* | GRAVID | F | negative |
| SLO1A4210 | 2008 | May | Osrednjeslovenska | *Myotis myotis* | GRAVID | F | negative |
| SLO1A4211 | 2008 | May | Osrednjeslovenska | *Myotis myotis* | GRAVID | F | negative |
| SLO1A0026 | 2008 | Jun | Osrednjeslovenska | *Pipistrellus kuhlii* | GRAVID | F | negative |
| SLO1A2146 | 2008 | Jul | Dolenjska | *Myotis daubentonii* | AD | M | negative |
| SLO1A2148 | 2008 | Jul | Dolenjska | *Myotis daubentonii* | AD | M | negative |
| SLO1A2149 | 2008 | Jul | Dolenjska | *Myotis daubentonii* | LACTATING | F | negative |
| SLO1A2151 | 2008 | Jul | Dolenjska | *Myotis daubentonii* | AD | M | negative |
| SLO1A4279 | 2008 | Jul | Dolenjska | *Myotis myotis* | AD | M | negative |
| SLO1A4291 | 2008 | Jul | Pomurska | *Myotis myotis* | JUV | M | negative |
| SLO1A4271 | 2008 | Jul | Dolenjska | *Myotis myotis* | AD | F | negative |
| SLO1A4273 | 2008 | Jul | Dolenjska | *Myotis myotis* | JUV | F | positive |
| SLO1A4275 | 2008 | Jul | Dolenjska | *Myotis myotis* | JUV | M | negative |
| SLO1A4277 | 2008 | Jul | Dolenjska | *Myotis myotis* | LACTATING | F | negative |
| SLO1A4265 | 2008 | Jul | Dolenjska | *Myotis myotis* | AD | F | negative |
| SLO1A4266 | 2008 | Jul | Dolenjska | *Myotis myotis* | LACTATING | F | negative |
| SLO1A4268 | 2008 | Jul | Dolenjska | *Myotis myotis* | LACTATING | F | negative |
| SLO1A4269 | 2008 | Jul | Dolenjska | *Myotis myotis* | JUV | F | negative |
| SLO1A4280 | 2008 | Jul | Pomurska | *Myotis myotis* | LACTATING | F | negative |
| SLO1A4281 | 2008 | Jul | Pomurska | *Myotis myotis* | JUV | F | negative |
| SLO1A4282 | 2008 | Jul | Pomurska | *Myotis myotis* | LACTATING | F | negative |
| SLO1A4283 | 2008 | Jul | Pomurska | *Myotis myotis* | JUV | M | negative |
| SLO1A4284 | 2008 | Jul | Pomurska | *Myotis myotis* | JUV | M | negative |
| SLO1A4285 | 2008 | Jul | Pomurska | *Myotis myotis* | LACTATING | F | negative |
| SLO1A4286 | 2008 | Jul | Pomurska | *Myotis myotis* | LACTATING | F | negative |
| SLO1A2144 | 2008 | Jul | Dolenjska | *Myotis mystacinus* | AD | M | negative |
| SLO1A2159 | 2008 | Aug | Dolenjska | *Myotis daubentonii* | AD | M | negative |
| SLO1A2161 | 2008 | Aug | Dolenjska | *Myotis daubentonii* | AD | M | negative |
| SLO1A2162 | 2008 | Aug | Dolenjska | *Myotis daubentonii* | JUV | F | negative |
| SLO1A2163 | 2008 | Aug | Dolenjska | *Myotis daubentonii* | JUV | M | negative |
| SLO1A2164 | 2008 | Aug | Dolenjska | *Myotis daubentonii* | LACTATING | F | negative |
| SLO1A2168 | 2008 | Aug | Dolenjska | *Myotis daubentonii* | LACTATING | F | negative |
| SLO1A2171 | 2008 | Aug | Dolenjska | *Myotis daubentonii* | JUV | M | negative |
| SLO1A2172 | 2008 | Aug | Dolenjska | *Myotis daubentonii* | JUV | F | negative |
| SLO1A2165 | 2008 | Aug | Dolenjska | *Myotis daubentonii* | JUV | F | negative |
| SLO1A0047 | 2008 | Aug | Dolenjska | *Pipistrellus pygmaeus* | AD | M | negative |
| SLO1A2152 | 2008 | Aug | Osrednjeslovenska | *Myotis daubentonii* | JUV | F | negative |
| SLO1A2153 | 2008 | Aug | Osrednjeslovenska | *Myotis daubentonii* | AD | M | negative |
| SLO1A2154 | 2008 | Aug | Osrednjeslovenska | *Myotis daubentonii* | JUV | F | negative |
| SLO1A2155 | 2008 | Aug | Osrednjeslovenska | *Myotis daubentonii* | AD | M | negative |
| SLO1A2156 | 2008 | Aug | Osrednjeslovenska | *Myotis daubentonii* | AD | M | negative |
| SLO1A2157 | 2008 | Aug | Podravska | *Myotis daubentonii* | JUV | F | negative |
| SLO1A2158 | 2008 | Aug | Podravska | *Myotis mystacinus* | JUV | M | negative |
| SLO1A4356 | 2008 | Sep | Podravska | *Myotis myotis* | LACTATING | F | negative |
| SLO1A4357 | 2008 | Sep | Podravska | *Myotis myotis* | AD | M | negative |
| SLO1A4361 | 2008 | Sep | Podravska | *Myotis myotis* | JUV | F | negative |
| SLO1A0087 | 2008 | Sep | Primorska | *Rhinolophus hipposideros* | JUV | M | negative |
| SLO1A0088 | 2008 | Sep | Primorska | *Rhinolophus hipposideros* | JUV | F | negative |
| SLO1A0089 | 2008 | Sep | Primorska | *Rhinolophus hipposideros* | LACTATING | F | negative |
| SLO1A0062 | 2008 | Sep | Dolenjska | *Rhinolophus hipposideros* | LACTATING | F | negative |
| SLO1A0063 | 2008 | Sep | Dolenjska | *Rhinolophus hipposideros* | LACTATING | F | negative |
| SLO1A0064 | 2008 | Sep | Dolenjska | *Rhinolophus hipposideros* | LACTATING | F | negative |
| SLO1A0065 | 2008 | Sep | Dolenjska | *Rhinolophus hipposideros* | LACTATING | F | negative |
| SLO1A4362 | 2008 | Sep | Podravska | *Myotis myotis* | AD | M | negative |
| SLO1A0048 | 2008 | Sep | Dolenjska | *Rhinolophus hipposideros* | LACTATING | F | negative |
| SLO1A0050 | 2008 | Sep | Dolenjska | *Rhinolophus hipposideros* | JUV | M | positive |
| SLO1A0052 | 2008 | Sep | Dolenjska | *Rhinolophus hipposideros* | JUV | M | negative |
| SLO1A0053 | 2008 | Sep | Dolenjska | *Rhinolophus hipposideros* | JUV | F | negative |
| SLO1A0054 | 2008 | Sep | Dolenjska | *Rhinolophus hipposideros* | JUV | F | negative |
| SLO1A0055 | 2008 | Sep | Dolenjska | *Rhinolophus hipposideros* | JUV | F | negative |
| SLO1A0081 | 2008 | Sep | Osrednjeslovenska | *Rhinolophus hipposideros* | LACTATING | F | negative |
| SLO1A0082 | 2008 | Sep | Osrednjeslovenska | *Rhinolophus hipposideros* | LACTATING | F | negative |
| SLO1A0083 | 2008 | Sep | Osrednjeslovenska | *Rhinolophus hipposideros* | LACTATING | F | negative |
| SLO1A0084 | 2008 | Sep | Osrednjeslovenska | *Rhinolophus hipposideros* | LACTATING | F | negative |
| SLO1A0085 | 2008 | Sep | Osrednjeslovenska | *Rhinolophus hipposideros* | LACTATING | F | negative |
| SLO1A0057 | 2008 | Sep | Dolenjska | *Rhinolophus hipposideros* | JUV | F | negative |
| SLO1A0058 | 2008 | Sep | Dolenjska | *Rhinolophus hipposideros* | JUV | M | negative |
| SLO1A0060 | 2008 | Sep | Dolenjska | *Rhinolophus hipposideros* | JUV | M | negative |
| SLO1A0066 | 2008 | Sep | Podravska | *Rhinolophus hipposideros* | LACTATING | F | negative |
| SLO1A0067 | 2008 | Sep | Podravska | *Rhinolophus hipposideros* | LACTATING | F | negative |
| SLO1A0068 | 2008 | Sep | Podravska | *Rhinolophus hipposideros* | LACTATING | F | negative |
| SLO1A0069 | 2008 | Sep | Podravska | *Rhinolophus hipposideros* | LACTATING | F | negative |
| SLO1A0070 | 2008 | Sep | Podravska | *Rhinolophus hipposideros* | LACTATING | F | negative |
| SLO1A0071 | 2008 | Sep | Podravska | *Rhinolophus hipposideros* | JUV | F | negative |
| SLO1A0076 | 2008 | Sep | Osrednjeslovenska | *Rhinolophus hipposideros* | LACTATING | F | negative |
| SLO1A0078 | 2008 | Sep | Osrednjeslovenska | *Rhinolophus hipposideros* | LACTATING | F | negative |
| SLO1A0079 | 2008 | Sep | Osrednjeslovenska | *Rhinolophus hipposideros* | LACTATING | F | negative |
| SLO1A0080 | 2008 | Sep | Osrednjeslovenska | *Rhinolophus hipposideros* | LACTATING | F | negative |
| SLO1A2181 | 2008 | Oct | Osrednjeslovenska | *Pipistrellus nathusii* | JUV | F | negative |
| SLO1A2180 | 2008 | Oct | Osrednjeslovenska | *Myotis mystacinus s.lat.* | AD | M | negative |
| SLO1A2178 | 2008 | Oct | Osrednjeslovenska | *Myotis daubentonii* | AD | M | negative |
| SLO1A2179 | 2008 | Oct | Osrednjeslovenska | *Myotis daubentonii* | AD | M | negative |
| SLO1A2183 | 2008 | Oct | Primorska | *Rhinolophus hipposideros* | JUV | M | negative |
| SLO1A2184 | 2008 | Oct | Primorska | *Myotis daubentonii* | AD | M | negative |
| SLO1A2185 | 2008 | Oct | Primorska | *Pipistrellus nathusii* | AD | M | negative |
| SLO1A2186 | 2008 | Oct | Primorska | *Myotis daubentonii* | AD | M | negative |
| SLO1A2187 | 2008 | Oct | Primorska | *Myotis capaccinii* | JUV | F | negative |
| SLO1A2188 | 2008 | Oct | Primorska | *Myotis mystacinus* | LACTATING | F | negative |
| SLO1A2174 | 2008 | Sept | Osrednjeslovenska | *Myotis mystacinus* | JUV | F | negative |
| SLO1A2175 | 2008 | Sept | Osrednjeslovenska | *Myotis daubentonii* | AD | M | negative |
| SLO1A2176 | 2008 | Sept | Osrednjeslovenska | *Rhinolophus hipposideros* | JUV | M | negative |
| SLO1A2177 | 2008 | Sept | Osrednjeslovenska | *Myotis daubentonii* | AD | M | negative |
