# Supplementary material for: Identification of novel reassortant mammalian orthoreoviruses from bats in Slovenia
Source: BMC Vet Res. 2018 Sep 3;14:264. doi: 10.1186/s12917-018-1585-y (PMC6122641; doi:10.1186/s12917-018-1585-y)
Supplement: Supplementary file 3 — Bat and sample characteristics of the pooled guano samples. (DOCX 13 kb) [file 12917_2018_1585_MOESM3_ESM.docx]

| **Sample code** | **Year** | **Location** | **Bat species** | **Known status (n)** | | **MRV RNA** |
| --- | --- | --- | --- | --- | --- | --- |
|  |  |  |  | **Age** | **Sex** |  |
| SLO1A4708+4709 | 2012 | Osrednjeslovenska | *Eptesicus serotinus* | Adult (2) | F(2) | Negative |
| CKFF1201+1202 | 2012 | Dolenjska | *Eptesicus serotinus* | Adult (2) | F(2) | Negative |
| SLO1A4599+4600 | 2010 | Dolenjska | *Eptesicus serotinus* | Juvenile (2) | M(1), F(1) | Negative |
| SLO1A4602+4603 | 2010 | Dolenjska | *Eptesicus serotinus* | Juvenile (1), Lactating female (1) | M(1), F(1) | Positive |
| CKFF17-21,24,26 | 2010 | Podravska | *Miniopterus schreibersii* | Adult (2), Lactating female (5) | M(2), F(5) | Negative |
| SLO1A4587-4589 | 2010 | Podravska | *Myotis myotis* | Adult (1), Lactating female (2) | F(3) | Negative |
| SLO1A4591, 4597, 4598 | 2010 | Podravska | *Myotis myotis* | Adult (1), Lactating female (2) | M(1), F(2) | Negative |
| SLO1A2990+2998 | 2010 | Unknown | Unknown | Unknown (2) | Unknown | Negative |

**Additional file 3:** Bat and sample characteristics of the pooled guano samples.
